# A Web-Based Distance Education Course in Nutrition in Public Health: Case study

**DOI:** 10.2196/jmir.3.2.e16

**Published:** 2001-04-19

**Authors:** Dirce M Sigulem, Tania B Morais, Lilian Cuppari, Sylvia C.C Franceschini, Silvia E Priore, Kátia G Camargo, Reinaldo Gimenez, Viviane Bernardo, Daniel Sigulem

**Affiliations:** ^1^Post-graduate Course in NutritionFederal University of São Paulo Medical SchoolBrazil; ^2^Department of Nutrition and HealthFederal University of ViçosaBrazil; ^3^Department of Health InformaticsFederal University of São Paulo Medical SchoolBrazil

**Keywords:** Internet, Curriculum, Nutrition, Education, Distance, Education, Medical, Continuing, Questionnaires, Program Evaluation

## Abstract

**Background:**

Strict work timetables, personal and professional duties or an inability to be absent from home or work can all represent major constraints for those wishing to improve their professional skills. Within this context, the World Wide Web can allow people to conveniently follow distance courses from their homes.

**Objective:**

To present an experience in the use of the Web in the continuing education of healthcare professionals in Brazil.

**Methods:**

A Web-based distance education course in nutrition in public health was developed. The methodology was an adaptation of both problem-based and task-based learning. At the end of the course an evaluation questionnaire which covered the course's contents, the educational methodology and resources, the duration and schedule, and the use of the Web as a tool for distance education was given to the students.

**Results:**

There were 83 on line registrations from 13 states, 73 of the applicants were female, 62 had a degree in nutrition and 18 were physicians. From these; eleven students from ten states were chosen: nine female nutritionists, two female physicians, and one male physician. Seven students completed the course, took and passed the final exam. Of the other four students, two failed to follow the schedule, one had health problems, and one did not obtain the minimal score for sitting the final exam. The students had a mean age of 35, and a mean of ten years in practice. They all stated that they were unable to attend a regular course, even though they felt that they needed to improve their professional skills. Most of them studied seven days a week for between two and four hours a day. The students also felt that their professional skills had improved and each reported having made changes in their practice as a result of their participation. The students approved of the course's contents, methodology and resources, however they were divided about its duration. The Web as a tool in distance education was approved by the students. If it was not for the Web they could not have taken part in a continuing education program. All students said they would attend another virtual course, if available. Even though most of them did not have difficulty adapting to the virtual environment, they did feel that an adaptation period would be of value.

**Conclusions:**

A Web-based course may be more effective than other distance education methodologies because it is more interactive and dynamic. On-line material can be constantly reviewed and updated, and the students can have the opportunity to submit commentaries or questions directly to the teaching staff. A Web-based course also allows the students to go beyond the course content as they learn how to search and take advantage of the huge resources of information available on the Internet.

## Introduction

Continuous education is vital in the health sciences field due to the huge amounts of new data generated by the rapid growth of knowledge in the area. This gives rise to new challenges, not only for graduate professionals, but also for universities, which have to extend their role to continuous education and not be restricted solely to the instruction of undergraduate and postgraduate students.

Evidence indicates that traditional college environments deprive a large group of people from the opportunity to enhance their professional skills. In order to accommodate this group, universities have established a wide range of alternative options such as evening courses, correspondence courses, cassette/videotape learning packages and tele-courses. However, the needs of people already practicing in their fields are so unique that they are barely met, even by these educational methods. Strict work timetables, personal and professional duties or an inability to be absent from home or work can all represent major constraints for those wishing to improve their professional skills.

Within this context, the World Wide Web, as a relatively low cost tool for the democratization and dissemination of knowledge [1], can play a revolutionary role by allowing students to conveniently follow distance courses from their homes.

In Brazil, the potential usefulness of the Web is accentuated by the country's size and the uneven geographical distribution of the universities, which are mainly concentrated within the most developed regions. Furthermore, regional economic dissimilarities have lead to a shortage of human resources within the less developed regions, heightening the importance of measures that favor the development of the few professionals who are working in these areas.

Aware of this situation, the Federal University of São Paulo Medical School developed and produced a distance course in Nutrition in Public Health using the World Wide Web.

The main goals of this project were:

- to provide professionals in various regions of Brazil with the latest knowledge in the field of Nutrition in Public Health;
- to qualify the professionals to adequately diagnose and solve the major problems related to Nutrition in Public Health in Brazil; and
- to familiarize the professionals with the main computing resources available to assist them in this learning process.

In this article, an educational model of Web-based learning and its resources is described. The positive aspects and the restrictions of the model as well as our personal experiences with the implementation of a distance education course are discussed.

## Methods

In 1997, the Postgraduate Program in Nutrition and the Department of Health Informatics developed a distance course, to be delivered via the Internet, for specialization in Nutrition in Public Health.

In Brazil, one must pursue a postgraduate degree in order to specialize within a specific field. Pursuing a postgraduate degree is more demanding than partaking in other continuing education courses because it aims to improve the performance of its graduates in professional activity in a specific field that requires particular skills. Specialization also facilitates promotion for government employees and is a prerequisite for entry into a Master's degree program. Specialization courses are regulated by the Ministry of Education and can only be offered by universities accredited by it. This was the first project for a distance Web-based course in the health sciences field submitted to, and approved by, the Ministry. The project covered the course format, the goals, the schedule, the number of credits, the number of hours, and the students' evaluation and final exam. The course was also evaluated and approved by the Federal University of São Paulo which issued a degree certificate on completion of the course.

After the course had received these approvals, a link was placed on the University's web site (www.epm.br) to the Virtual Course of Nutrition in Public Health (www.virtual.epm.br/cursos/nutrica.htm). A range of information about the course is available on this page including a registration form which can be completed and submitted electronically. The course is free of charge and is currently run biannually.

There have been three editions of the course with ten places available in each one. The first edition, intended for nutritionists only, started in August 1997 and ended in December 1997. The second and third, which were intended for both nutritionists and doctors, ran from April to December 1998 and from March to July 2000, respectively. The course is delivered in Portuguese, although some of the documents used are in English or Spanish.

### The Courseware Development Model

#### The Educational Model: Problem-based and task-based learning

In 1997, it was necessary to develop an original methodology for the course as there was a shortage of educational models for use on the Web. A partnership between the faculty of University's Postgraduate Course in Nutrition, and the Education team of the Department of Health Informatics resulted in a computer and Web based learning design.

The faculty consisted of one experienced pediatrician (DMS) who was working on malnutrition and anemia in children, two nutritionists who were working on the nutritional status of pregnant women (SCCF) and adolescents (SEP), one nutritionist with clinical experience in nephrology (LC) and one graduate in biomedical sciences (TBM) with experience in food quality control.

The aims of the course guided the choice of the methodology, which was an adaptation of both problem-based and task-based learning [2,3,4]. Problem-based learning is one of the most appropriate methodologies for community-oriented instruction in health. Students are required to combine their knowledge of a range of areas and also to bring into consideration psychosocial elements which encourages them to take a wider and more critical view of the issues related to the health of the community [2]. One characteristic of this type of methodology is the stimulation of an active search for knowledge [2], which was in perfect accordance with one of the aims of the course.

**Figure 1:**
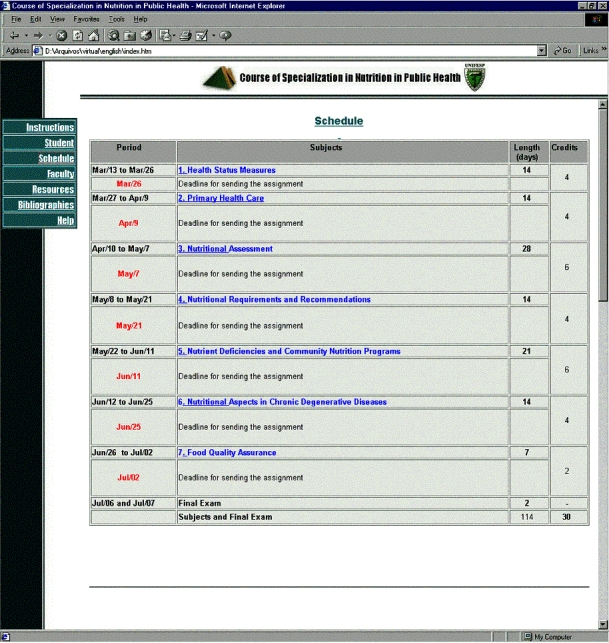
Course screenshot with course schedule

The faculty selected the seven main subjects which were to be developed during the course (Figure 1). For each of these subjects, a variable number of problems and tasks, of differing degrees of complexity, were prepared based on real day-to-day situations experienced by the teachers.

The number of credits given for each subject took into account its difficulty and ranged from 2 to 6 with a total of 30 credits in all. The structure of the course calendar also brought into consideration the difficulty of the tasks and the different periods allowed for their resolution (Figure 1).

#### Computer and Web resources

Taking into account that the students might not have easy access to libraries, as much information as possible was made available through the Web. This created an atmosphere of enjoyable, interactive learning.

**Figure 2:**
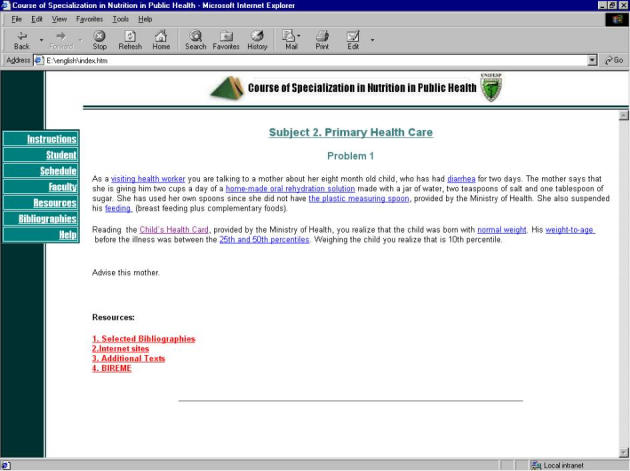
Course screenshot with a primary health care problem

For each problem there were direct links to references, sites of interest on the Internet, supplementary texts and links to BIREME (the Pan American Health Organization and World Health Organization health sciences information center for the Latin American and Caribbean area) (Figure 2).

**Figure 3:**
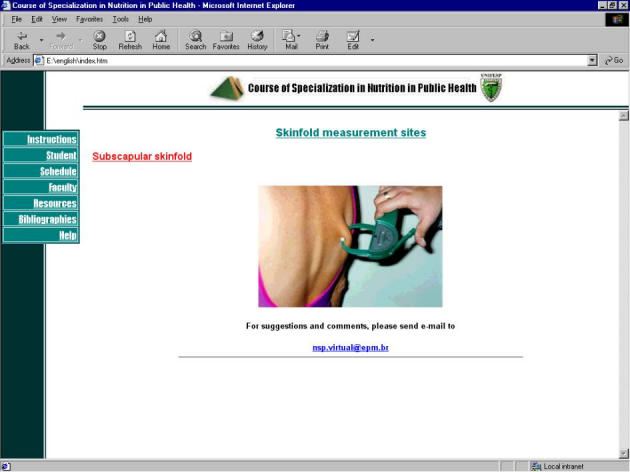
Course screenshot with an illustration from the image data bank of techniques and equipment

**Figure 4:**
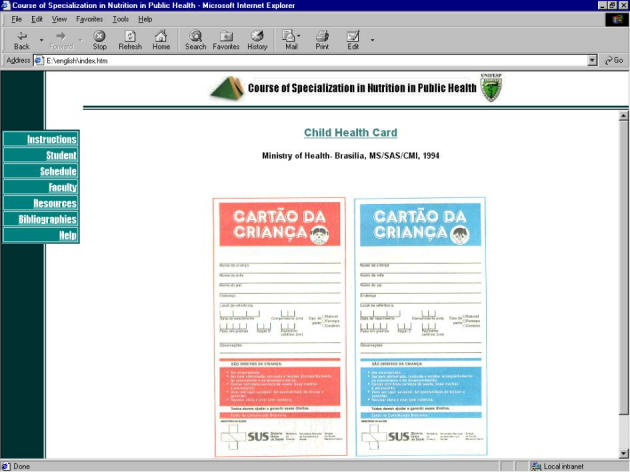
Course screenshot with Child Health Card

**Figure 5:**
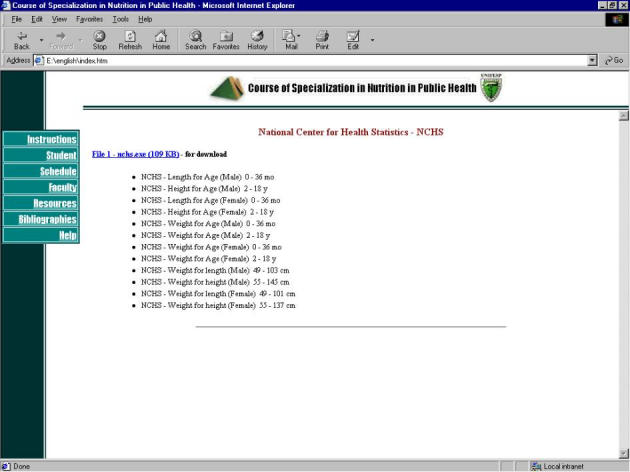
Course screenshot with tables from the National Center for Health Statistics (NCHS)

**Figure 6:**
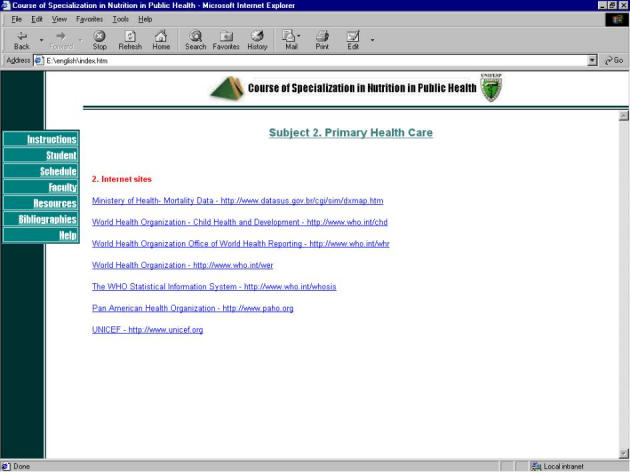
Course screenshot with external links

**Figure 7:**
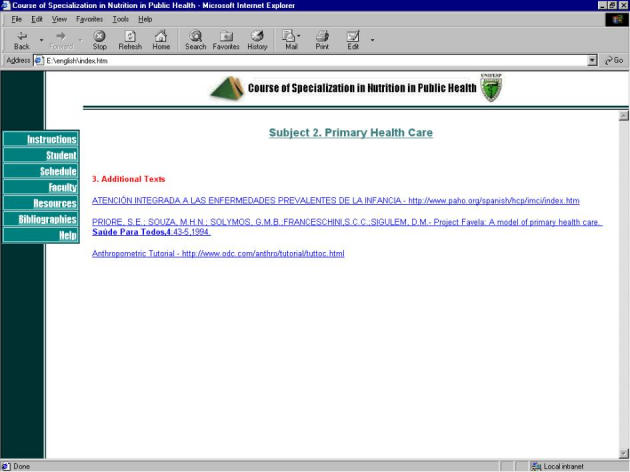
Course screenshot with links to supporting texts

Hyperlinks direct the students to various other resources such as a glossary, an image data bank of techniques and equipment (Figure 3), institutional material published by the Brazilian Ministry of Health (Figure 4), documents published by the World Health Organization (Changes in nutritional status, WHO, Belgium, 1983, which uses the tables of the National Center for Health Statistics, see Figure 5), as well as to other sites of interest (Figure 6) and supporting texts (Figure 7).

The pages and links were created using the software Namo Web Editor® 2.0. The image data bank, the institutional materials and the additional texts were created by first scanning and then editing the images using the software Aldus® Photo Styler® 2.0.

### The Implementation of the Course

#### Course structure

Following the Web-based registration of students interested in the course, the teaching staff selected the applicants who would take part. The aims of the course determined the most important selection criteria, which were the applicant's professional activity in Public Health and their geographical location, with preference being given to those applicants working in the less developed regions of Brazil. The applicants' curriculum vitae and a letter they had written justifying their interest in the course were also brought into consideration.

The selected students were given an electronic password which gave them access to the course at any time via a specially set-up web site. This web site also contained a list of the participants together with their photographs and e-mails.

The students worked individually on the solutions to the problems, although they could exchange information with each other, and with the teachers, by e-mail. The assignments were submitted electronically via e-mail using an attached file. The students were also supplied with a specific e-mail address at the Department of Health Informatics where they could seek assistance with any computer related problems.

#### Evaluation of the students

The students were evaluated through reports they wrote in response to the problems and tasks in each subject. In some subjects they also had to create posters, pamphlets and booklets to be used in health education. These materials were also considered for evaluation.

As the model used did not include pre-established, single answers for the tasks, grading was performed by all the instructors and the final grades were reached through a consensus. At the end of each subject, the teaching body wrote "Final Comments" in which a summary of the most important points and a correction of general concepts were made.

For each subject the students were awarded a mark in the range A to E. To calculate a score for the subject these marks were converted to values of 4 to 0, respectively, and this value was multiplied by the number of credits given for the subject. The scores in each subject were then summed to give an overall score, with a maximum of 120. A supervised final exam was taken at the University by those students with an overall score of at least 60 (50%). An accredited certificate was awarded to the students who passed the exam.

#### Methods of evaluation of the course

The first two courses were treated as preliminary experiences in the development of the proposed Web-based model. For these two courses a total of 60 on-line registrations were received, from which 20 students were chosen. Of these 20 students, a final total of 7 completed the courses and were approved. The main problem in the first course was the poor quality of Internet service which was available in Brazil in 1997, while in the second course there was a high dropout rate which was attributed to the course's long duration and the fact that many of the students did not work in the public health service and therefore did not feel that the course was relevant.

Through these experiences a satisfactory format for the course was developed. At the end of the third course a paper-based evaluation questionnaire was given to the students as they sat for the final exam. This questionnaire covered the course's contents, the educational methodology and resources, the course duration and schedule, the use of the Web as a tool for distance education, and the informatics support.

The dropout rate, the students' evolution throughout the course, and the changes in their practices were also bought into consideration in the overall course evaluation.

## Results

For the third course there were 83 on-line registrations from 13 states. Eighty-eight per cent (73/83) of the applicants were female. 75% (62/83) had a degree in nutrition and 22% (18/83) were physicians; the remaining three applicants had degrees in dentistry, biology and pharmacy. From these 83 registrations, eleven students from ten states were chosen: nine female nutritionists, two female physicians, and one male physician. The students who were not chosen either did not work in the public health system or lived in developed areas of the country. All the chosen students were government employees as this is the only class of employee that works in the public health service.

Seven students (64%) completed the course and took and passed the final exam. Of the other four students, two failed to follow the schedule (were not able to send the assignments in on the fixed day - see Figure 1) , one had health problems, and one did not reach the minimal score for sitting the final exam. The characteristics of the students who completed the course are shown in Table 1. Table 2 shows the students' self-reported study habits and Table 3 shows their evaluation of the course after the exam. The students' opinions of the Web as a tool for distance education are given in Table 4.

**Table 1:** Characteristics of the participants who passed the final exam

**Sex**	Female: 6 Male: 1
**Degree**	Nutrition: 6 Medicine: 1
**Mean age (years):**	35 (min.26-max.55)
**Mean years of Practice (years):**	10
**GovernmentEmployees:**	7

**Table 2:** Self-reported students study habits

**Time at which study started**	7:00 P.M. - 9:00 P.M. : 4 10:00 P.M. - 12:00 P.M. : 3
**Number of hours of study per day**	2 hours: 3 3 hours: 2 4 hours: 2
**Number of days of study per week**	seven days: 4 six days: 3
**Resources searched other than course resources**	other web sites: 5 books: 5 library: 1
**Methods used to save the course contents**	printed all the pages:2 just read on screen: 0 printed just the relevant pages: 3 saved to hard drive or diskettes: 2

**Table 3:** Course evaluation

**Contents**	good: 7 average: 0 bad: 0
**Methodology**	good: 7 average: 0 bad: 0
**Resources**	good: 6 average: 1 bad: 0
**Duration**	sufficient: 3 insufficient: 4
**Improvement of professional skills**	yes: 7 no: 0
**Practice changes;** ("Have you changed your professional practice as you learned new information during the course?")	yes: 7 no: 0

**Table 4:** Responses to the post-course survey among successful participants of the program

**Why did you attend a distance course on the Web?**	impossible to attend a regular course: 7 need to improve my professional skills: 7 lack of a college in my city: 6
**Did you have difficulty adapting to the virtual environment?**	yes: 1 no: 6
**Should the course have an initial period for adaptation to the virtual environment ?**	yes: 4 no: 3
**Would you attend another virtual course?**	yes: 7 no: 0

## Discussion

Although the use of the Web in continuing education is a relatively new practice [5,6,7], the high number of on-line applications that we received (a total of 143 in the three courses already delivered) showed the high demand for this kind of educational methodology. Indeed, many students applied for the course even though they didn't have the required profile. Nevertheless, as the course was very demanding both for the students and the faculty, only ten places per edition were made available, although less demanding courses could accommodate higher numbers of students.

For us, the most successful aspect of the course was the fact that it demonstrated that it is possible to reach professionals through the Web who would otherwise have been unable to take part in a continuing education program. The students were adults, with a mean age of 35, and were all experienced professionals with a mean of ten years in practice. They all stated that they were unable to attend a regular course, even though they felt that they needed to improve their professional skills. For most of them there was neither a college in their city nor an available library.

Possibly due to feelings of isolation, the students valued the opportunity the course gave them and demonstrated an extraordinary motivation. Working very hard; most of them studied seven days a week for between two and four hours a day. The methodology was found to be very satisfactory and the teaching staff was able to observe the positive evolution of the students as they developed their critical thinking and independent learning skills. The students also felt that their professional skills had improved and all of them reported having made changes in their practice as a result of their participation. This supports the idea that a model of continuing education that allows students to participate actively in the learning process, and that targets a need or deficiency perceived by them in their everyday practices, can result in better performance in examinations and an overall improvement in patient care outcomes [8]. Furthermore, as all the students were government employees, it can be expected that this had a beneficial effect on the public health care in the regions where they worked.

The students approved of the course's contents, methodology and resources, however they were divided on its duration. Four students thought that it was insufficient, although, from the eleven students who started the course, two were unable to follow the schedule. This issue of course duration is a controversial point and our previous experience has shown that a duration of more than four months increases the dropout rate. The faculty's feeling on this point was that the student's commitment to the course was more important than its duration.

It is worth emphasizing that the model used demanded a lot of work from the teaching staff and the amount of time spent in planning and delivery was greater than expected; a similar experience was reported by Chan et al [7]. One aspect that was especially time consuming was searching and evaluating external web sites. Furthermore, at the start of each course a complete revision of all the material was required as the external web sites often changed their structure, necessitating revision of all the links to them.

It is interesting to note that two of the teaching staff were based at another university about 700 kilometers away and were therefore also working at a distance. This further demonstrates the possibilities created by the Web for people in different locations to work on a common project including a distributed faculty for a training course.

The Web as a tool in distance education was enthusiastically approved by the students, all of who stated that they would attend another virtual course, if available. Indeed, if it were not for the Web none of the students would have been able to take part in a continuing education program.

Most of the students did not have difficulty adapting to the virtual environment, although they did feel that an adaptation period would be of value.

The main problems that were observed were related to such things as students' computers not meeting the minimum hardware or software requirements, students' lack of computer skills, low access speed to Internet service providers, and even temporary interruption of service. Also, some of the students had not expected to spend an average of 10 - 15 hours a week on the course-work, and were therefore unable to follow the course. A further restriction was that the didactic material used was necessarily limited to that which was not copyright, or was available for free on the Internet, or for which the author's permission for use was obtained.

Despite these limitations, the Web was shown to be a useful tool for distance education and should play an important role in the future of education, particularly in large countries with marked regional economic differences and unevenly distributed universities, like Brazil.

Our experience raised two issues that merit attention. The first is that it is important to define a precise profile of the target audience. The second is that a Web-based course may be more effective than other distance education methodologies because it is more interactive and dynamic. On-line material can be constantly reviewed and updated, and the students can have the opportunity to submit commentaries or questions directly to the teaching staff. A Web-based course also allows the students to go beyond the course content as they learn how to search and take advantage of the huge resources of information available on the Internet.

This educational model is already being reproduced by other departments at the Federal University of São Paulo including Dermatology, Ophthalmology and Orthopedics, and Traumatology.

## Multimedia Appendix

Downloadable Screenshots of the course
