# Supplementary material for: A Web-Based Distance Education Course in Nutrition in Public Health: Case study
Source: J Med Internet Res. 2001 Apr 19;3(2):e16. doi: 10.2196/jmir.3.2.e16 (PMC1761899; doi:10.2196/jmir.3.2.e16)

## Slide 1
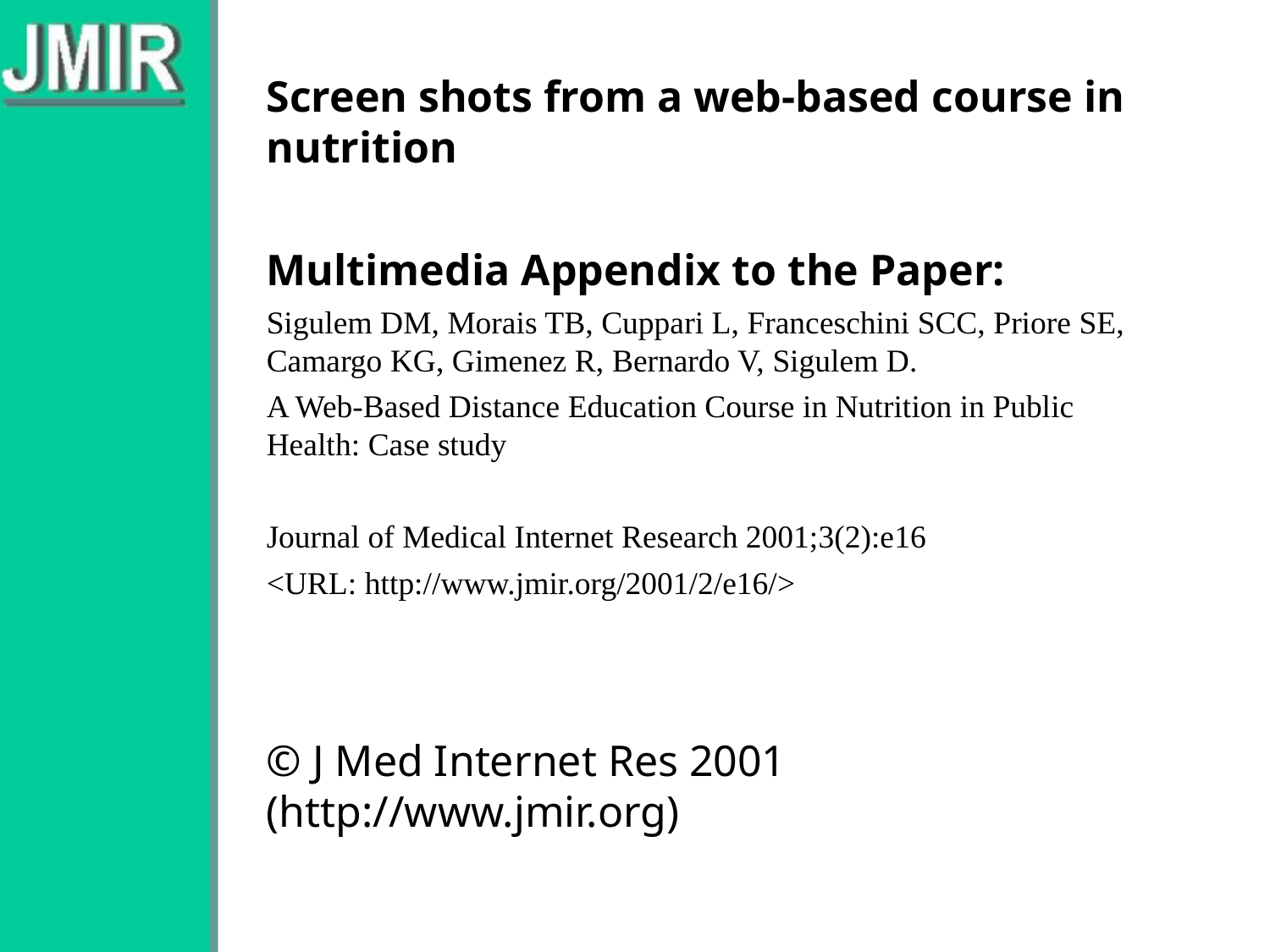

# Screen shots from a web-based course in nutrition
Multimedia Appendix to the Paper:
Sigulem DM, Morais TB, Cuppari L, Franceschini SCC, Priore SE, Camargo KG, Gimenez R, Bernardo V, Sigulem D.
A Web-Based Distance Education Course in Nutrition in Public Health: Case study
Journal of Medical Internet Research 2001;3(2):e16
<URL: http://www.jmir.org/2001/2/e16/>
© J Med Internet Res 2001 (http://www.jmir.org)

## Slide 2
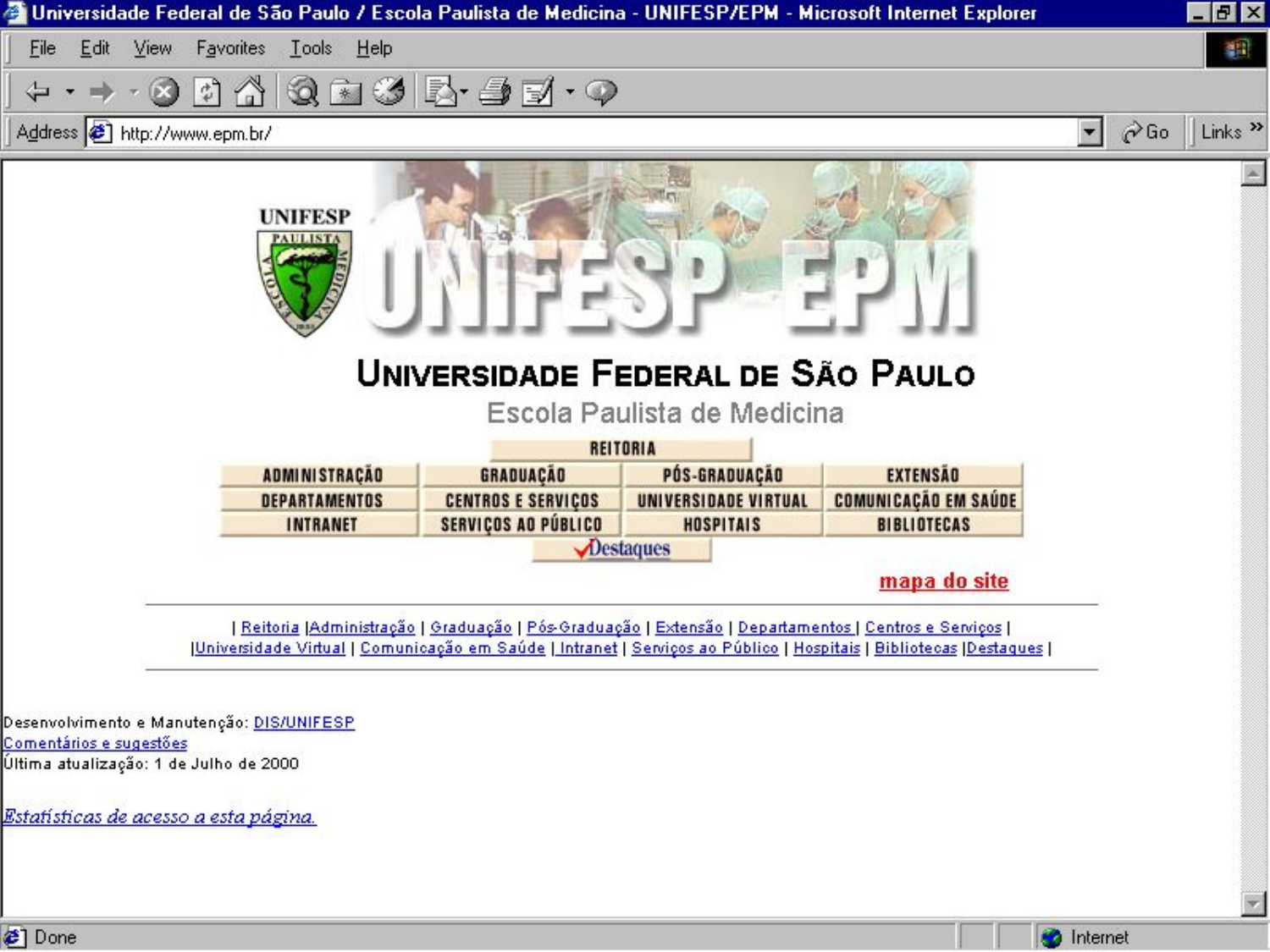

## Slide 3
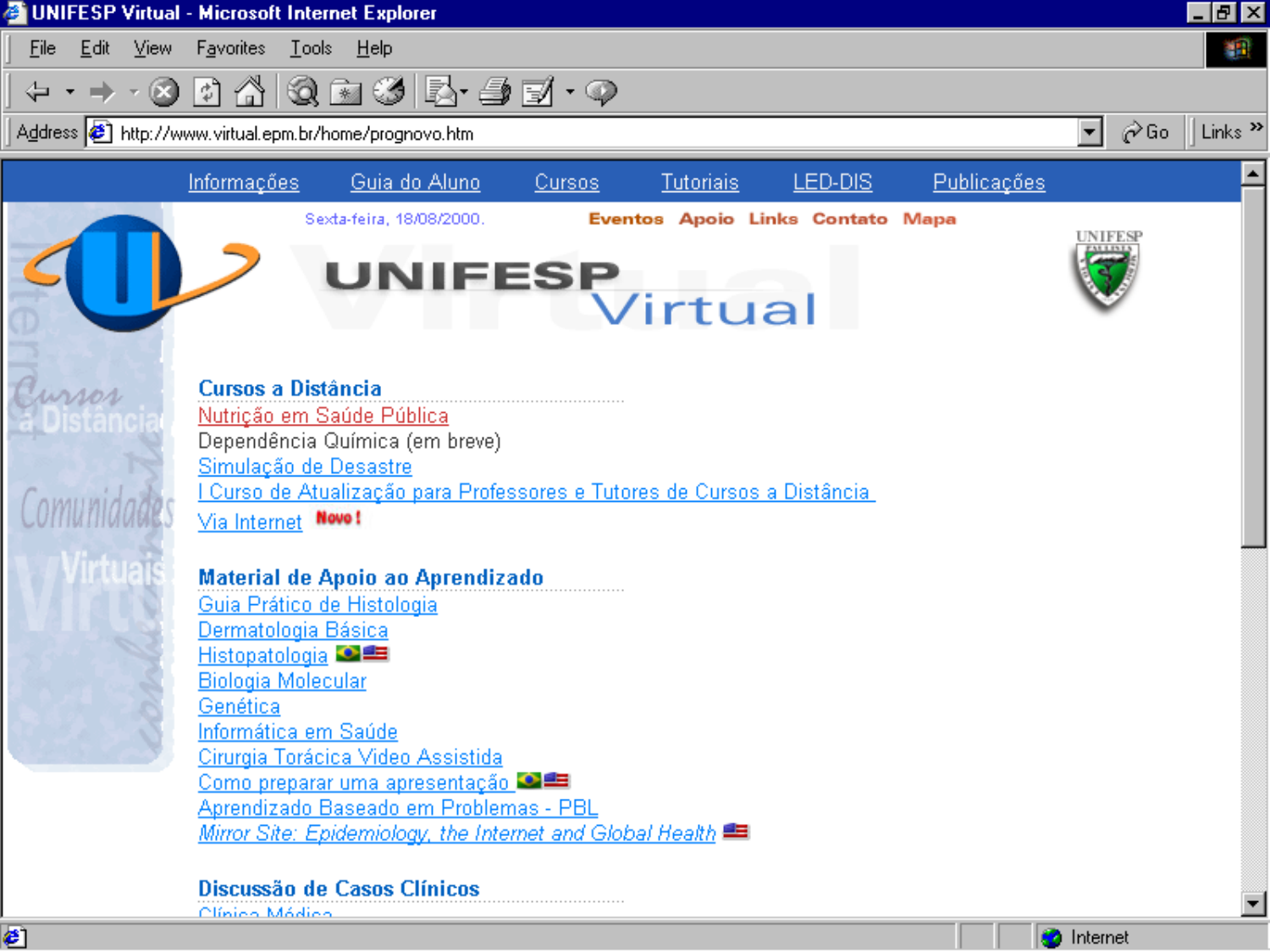

## Slide 4
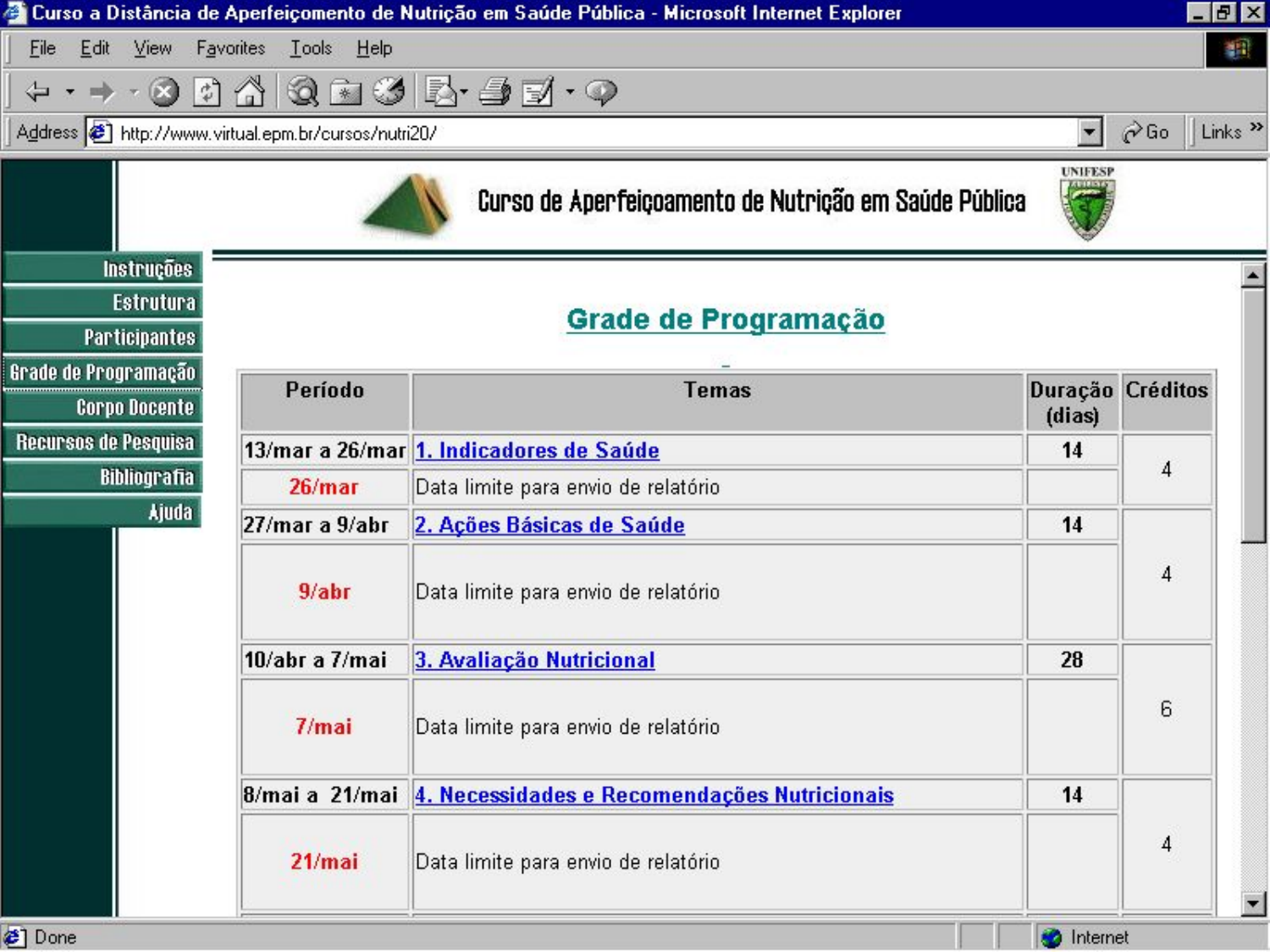

## Slide 5
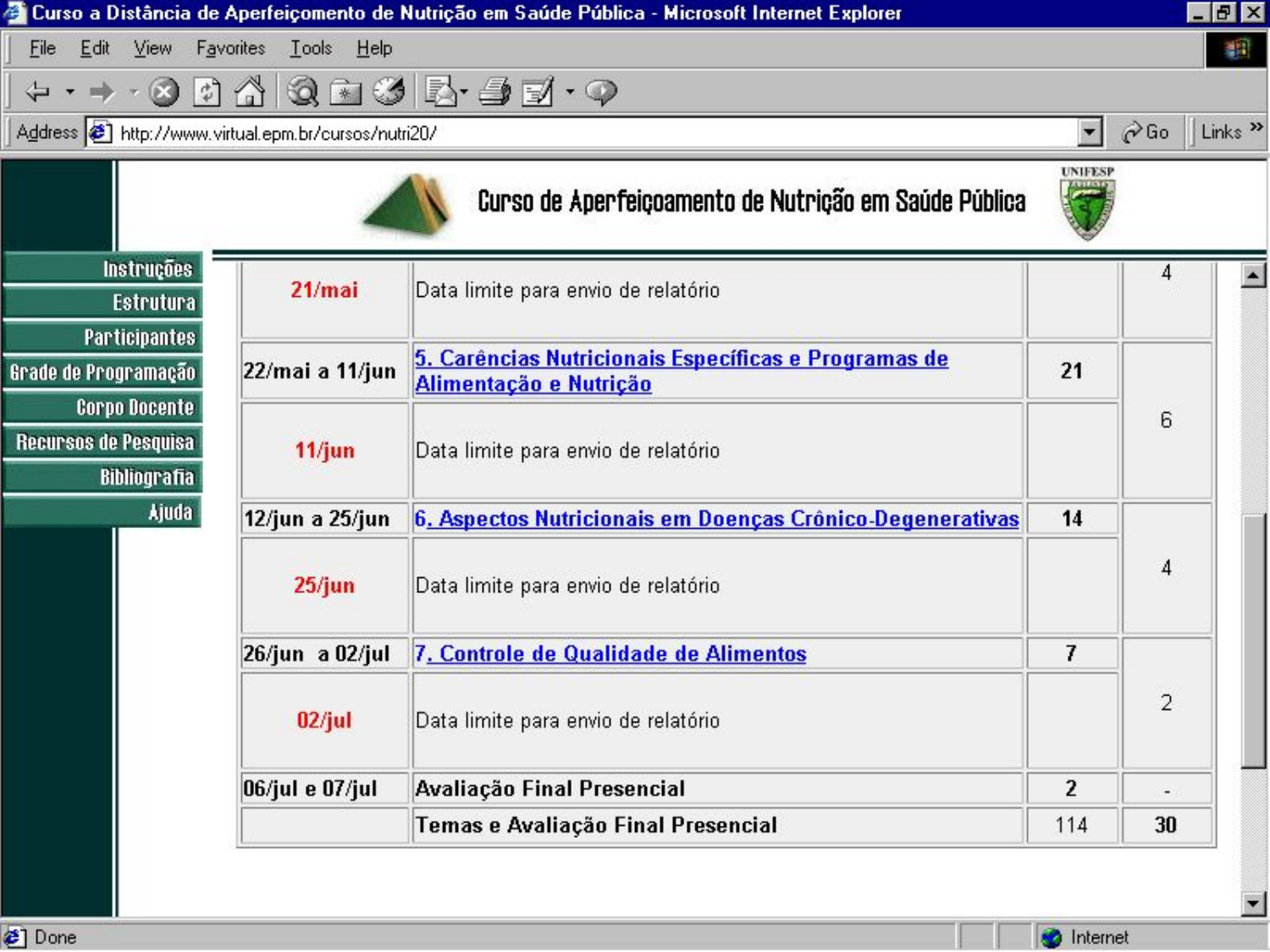

## Slide 6
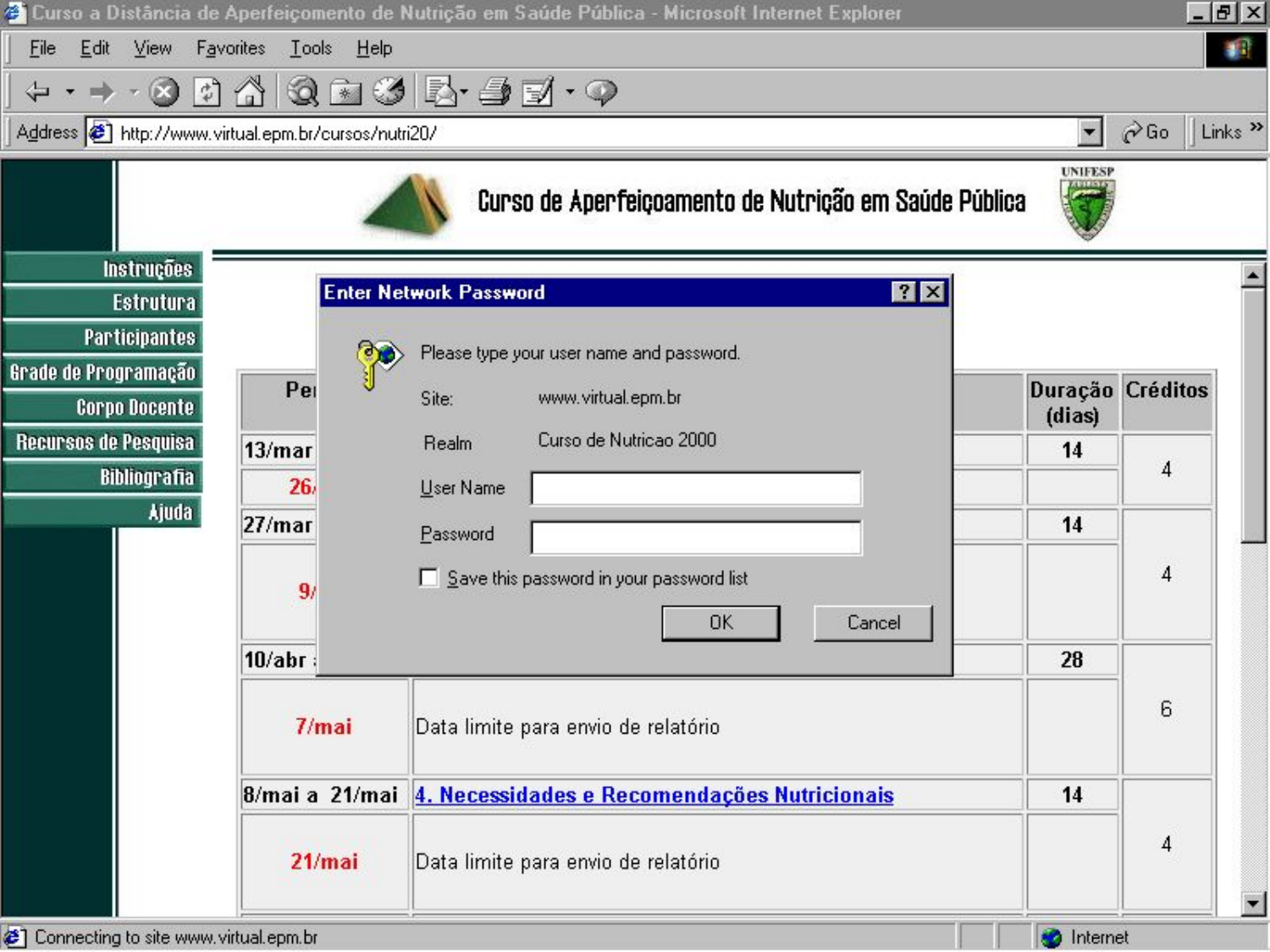

## Slide 7
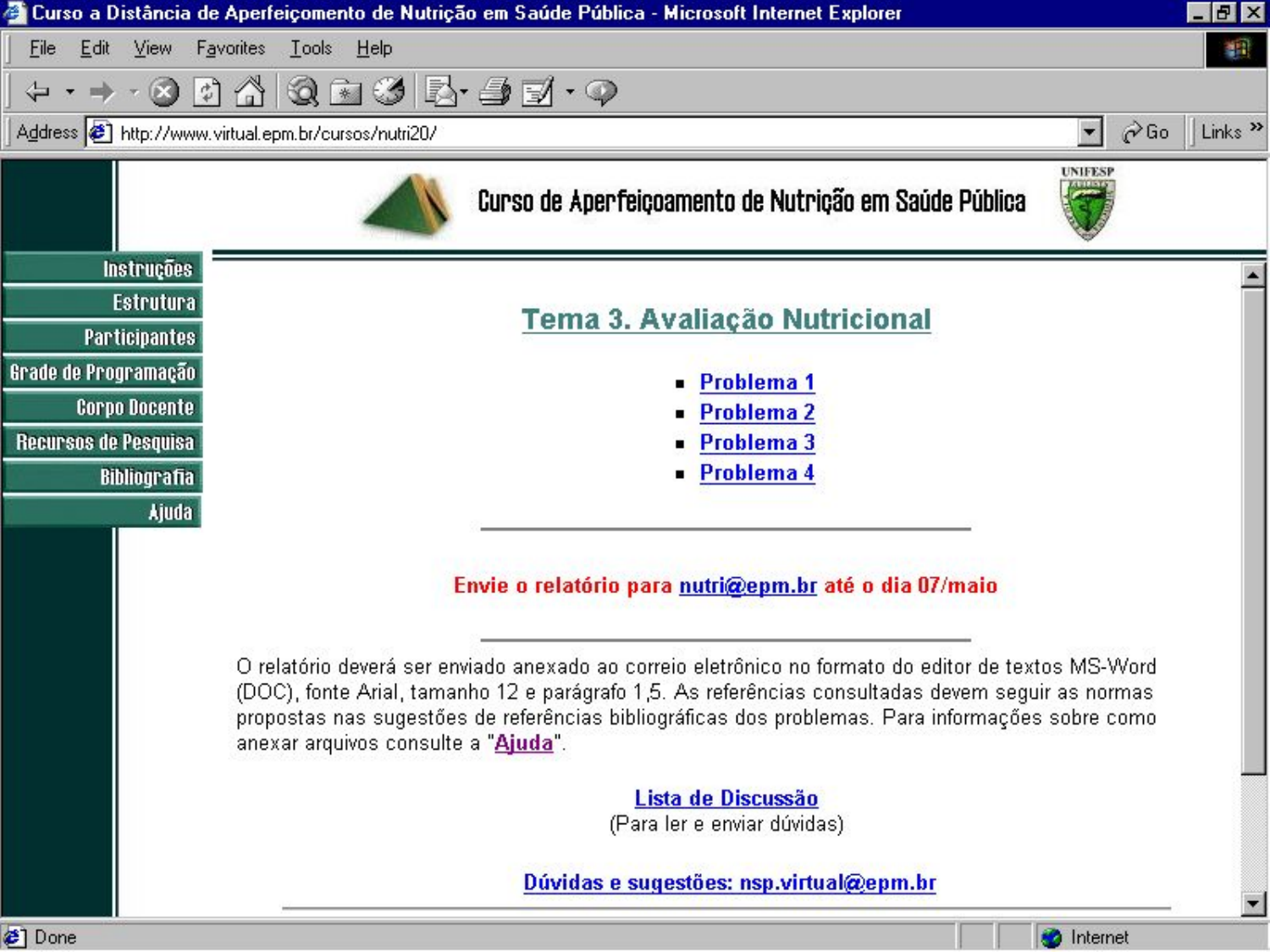

## Slide 8
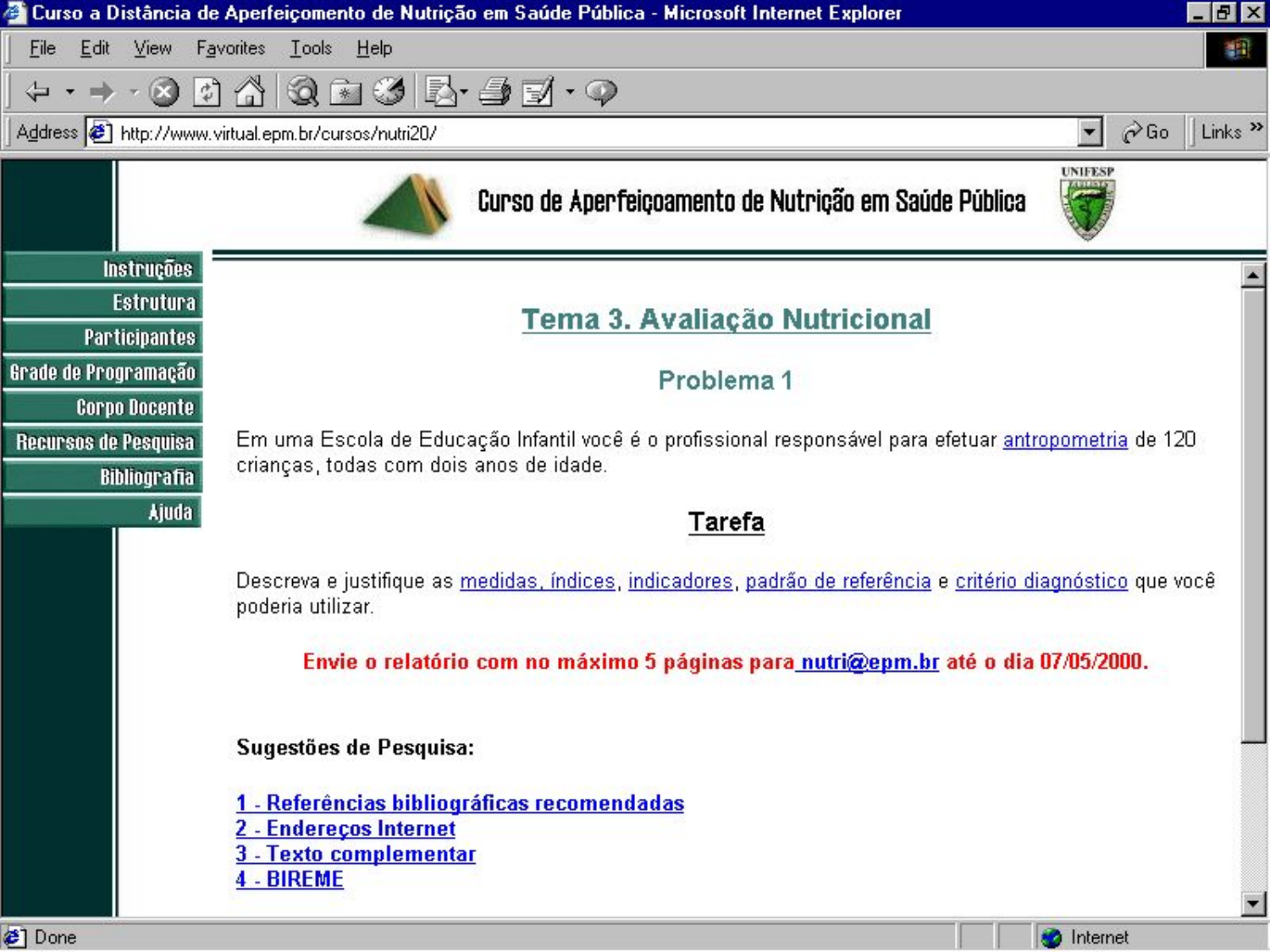

## Slide 9
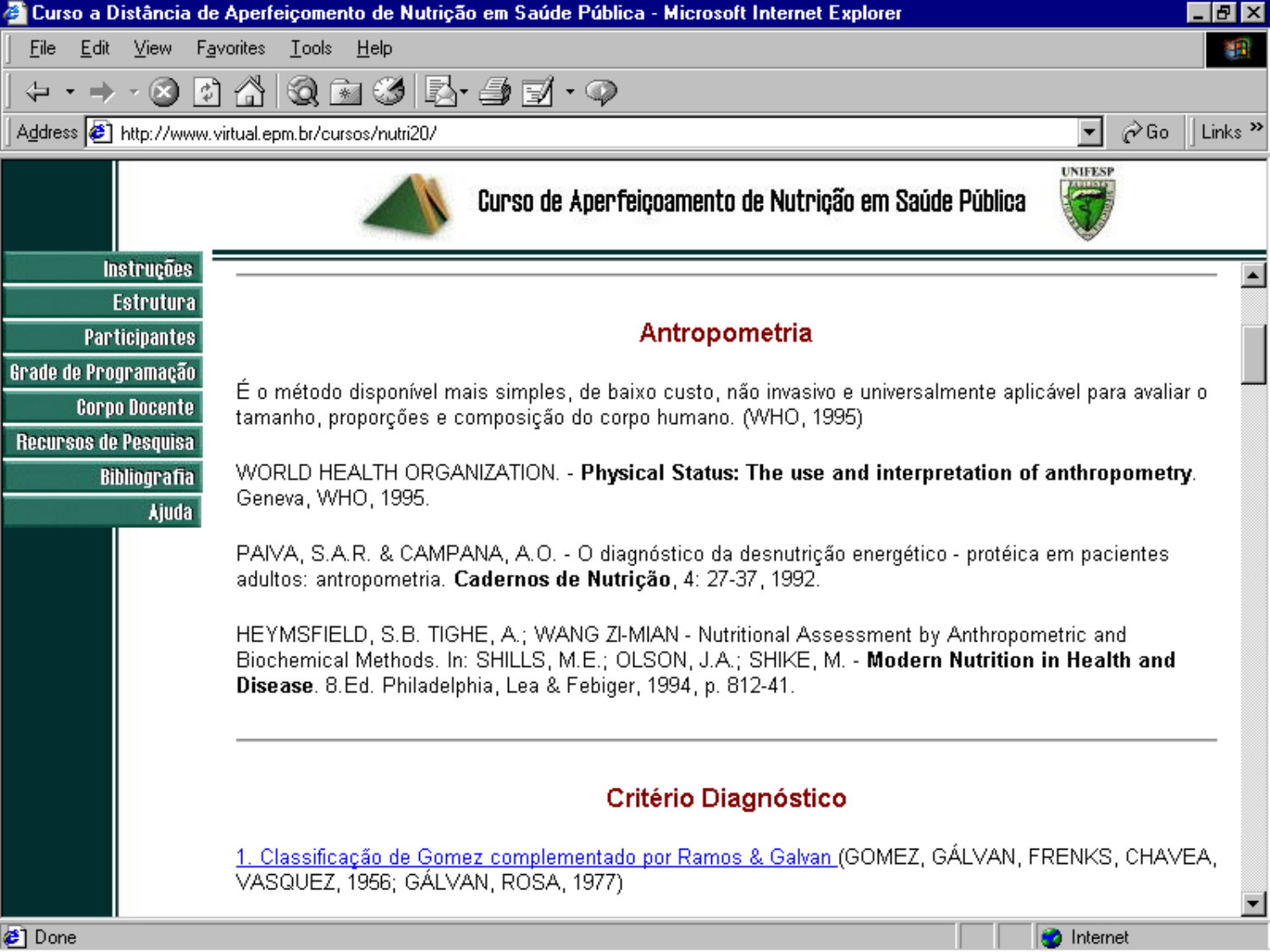

## Slide 10
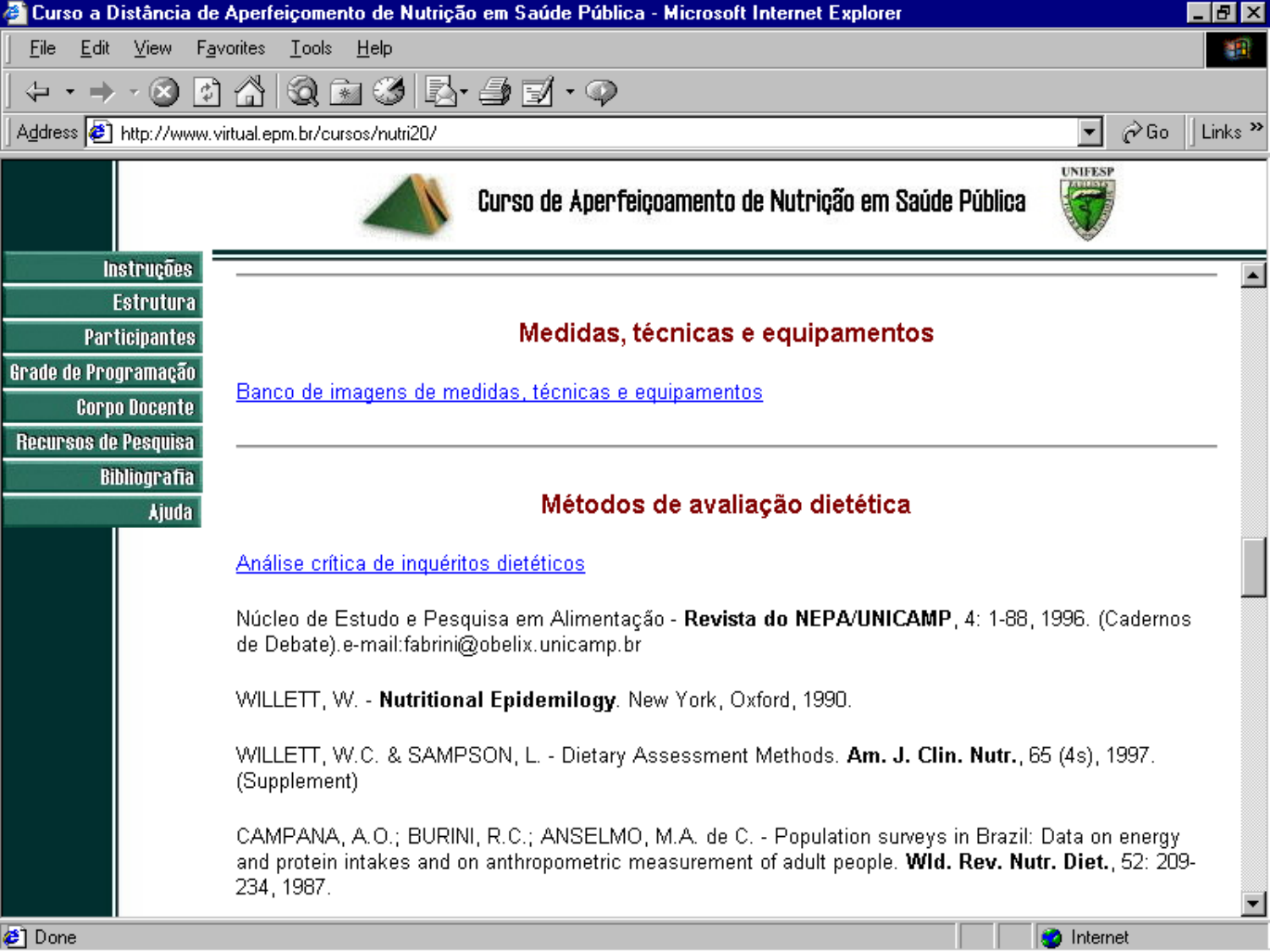

## Slide 11
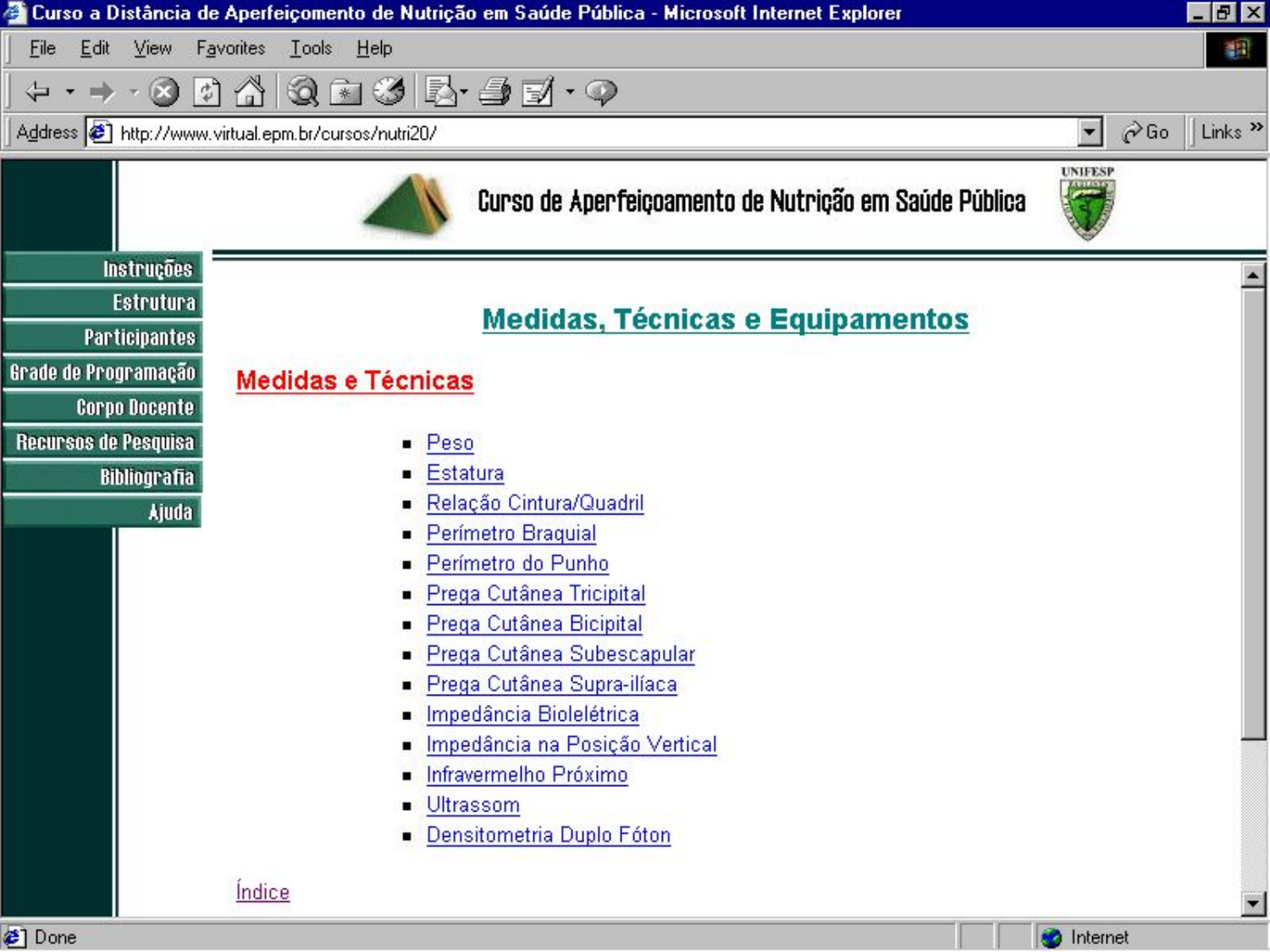

## Slide 12
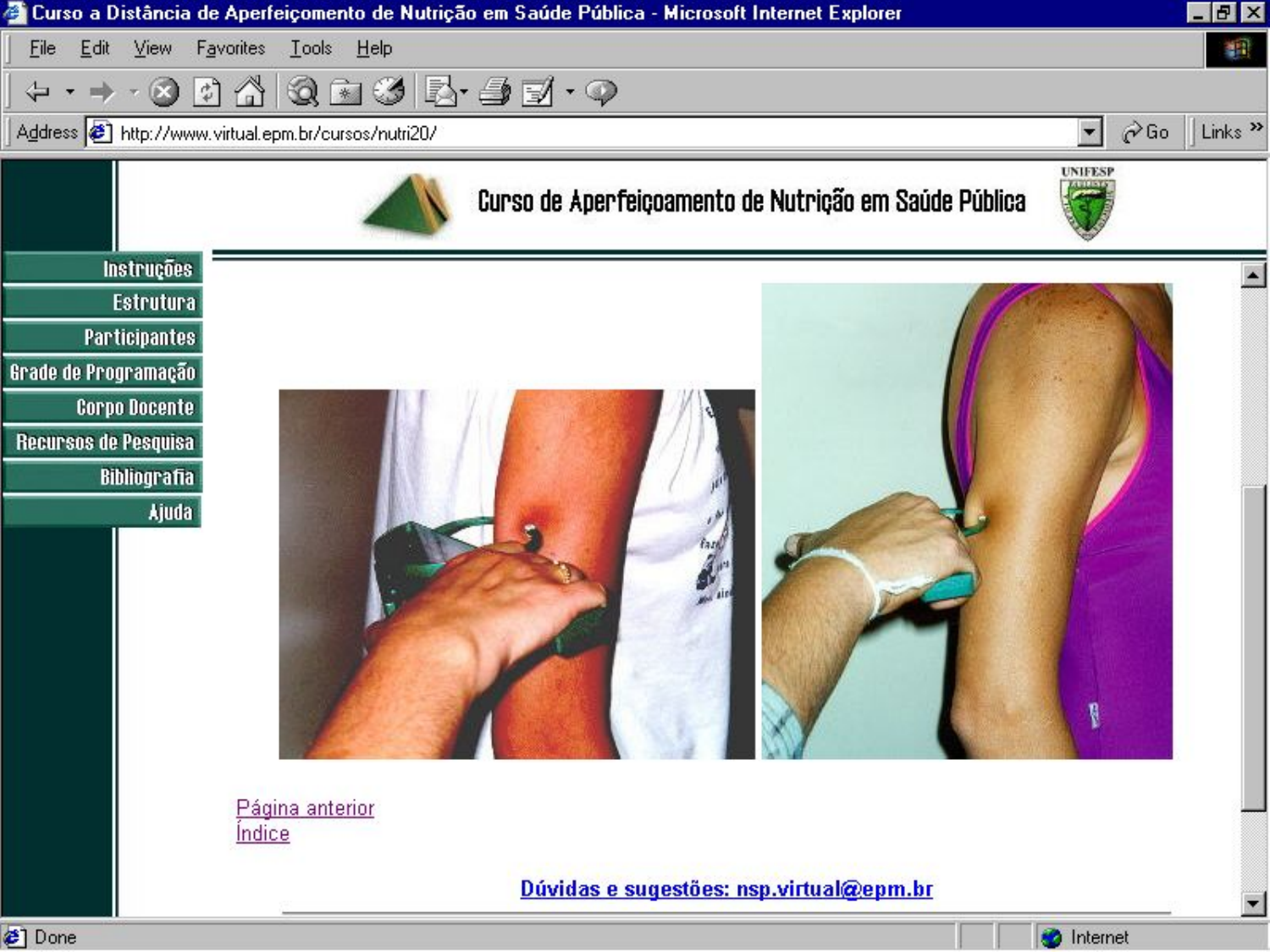

## Slide 13
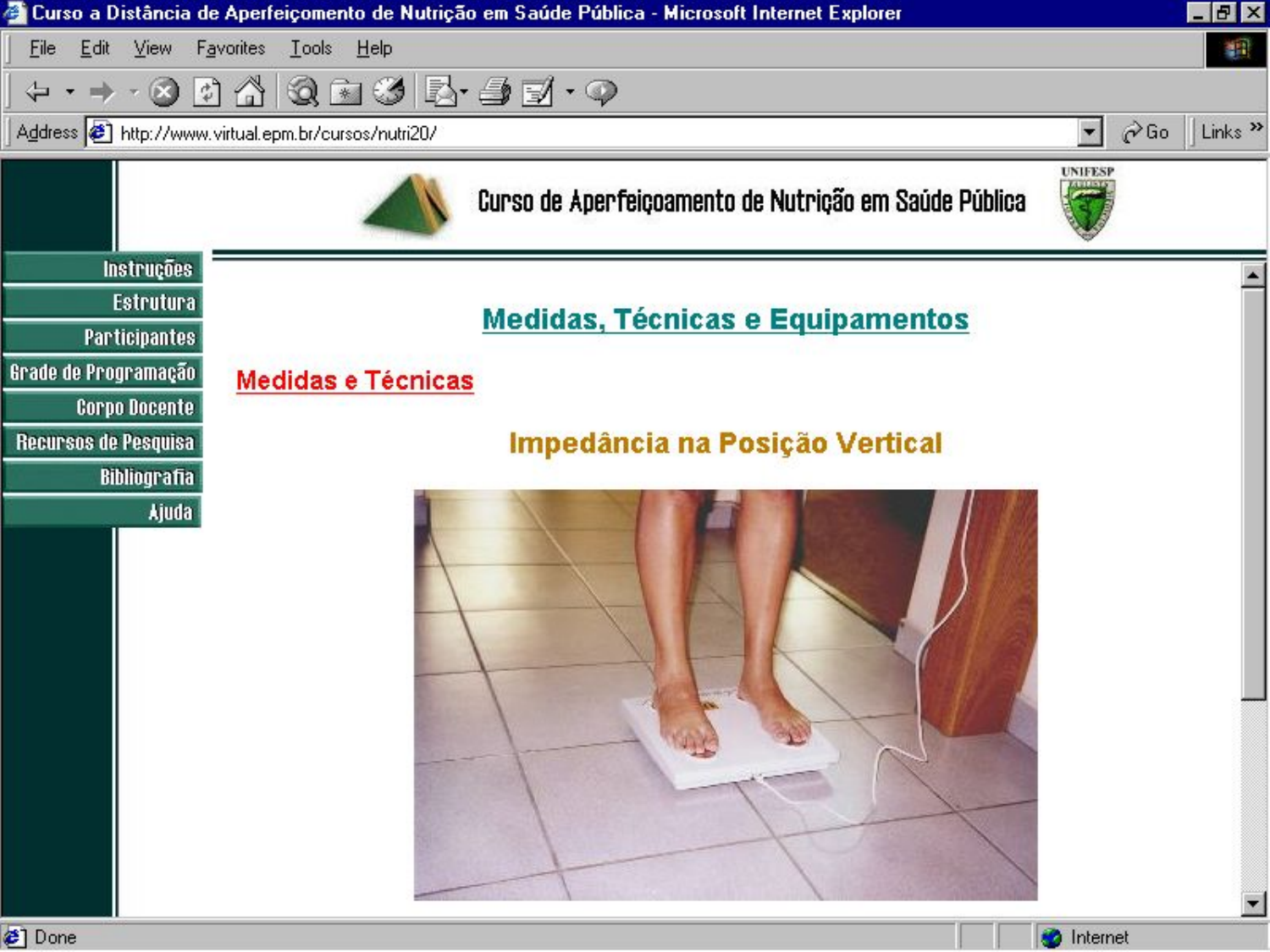

## Slide 14
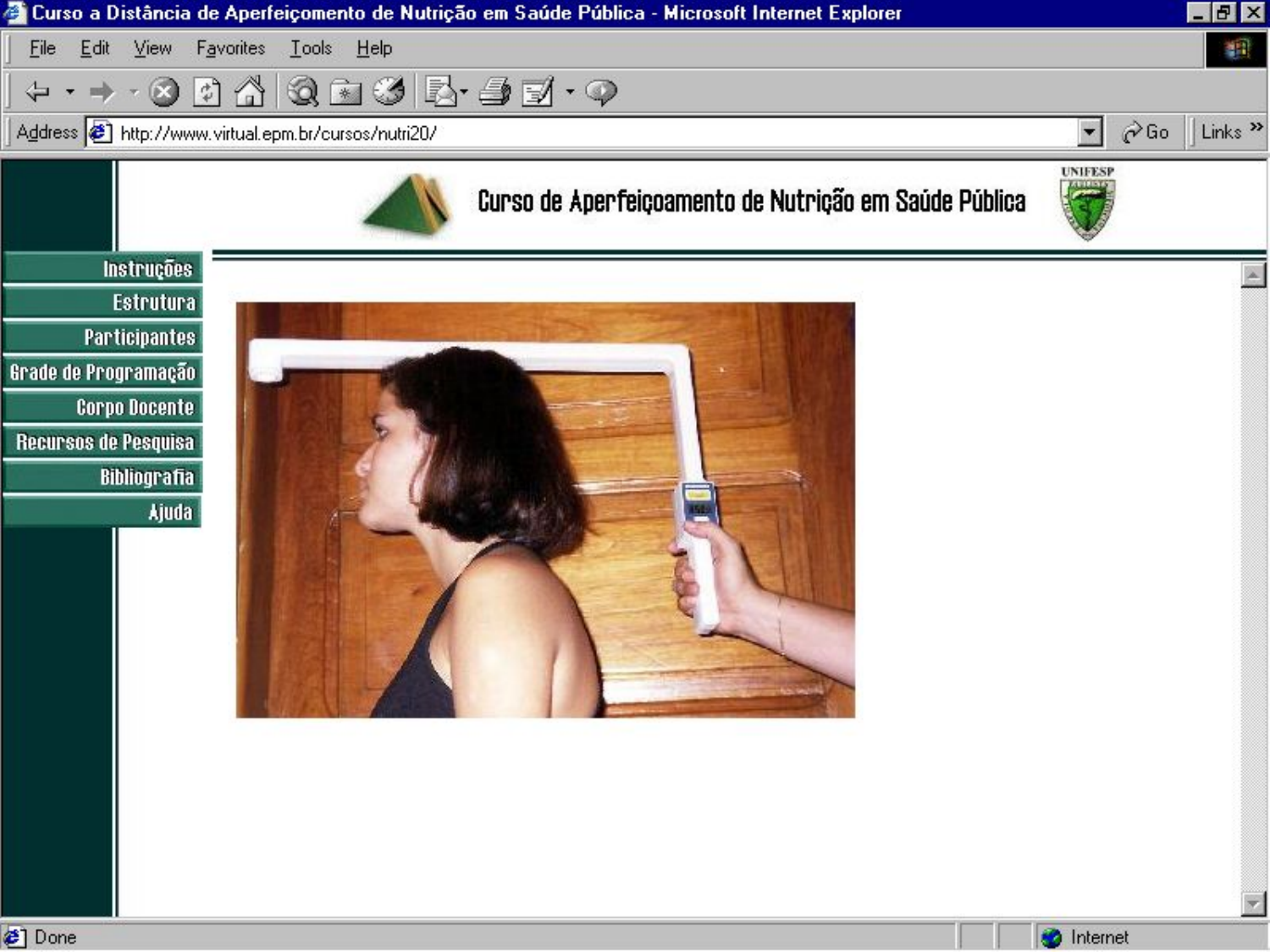

## Slide 15
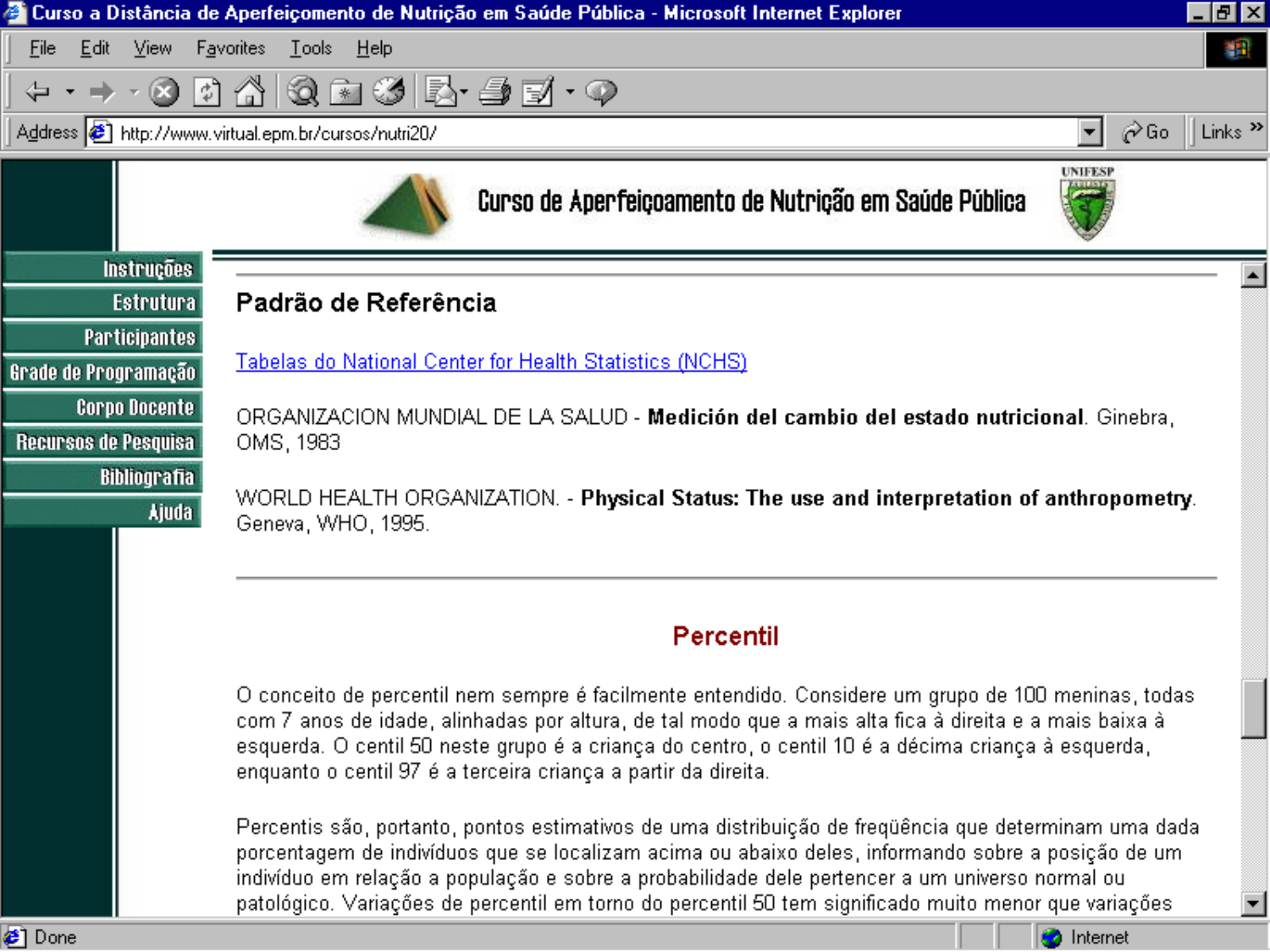

## Slide 16
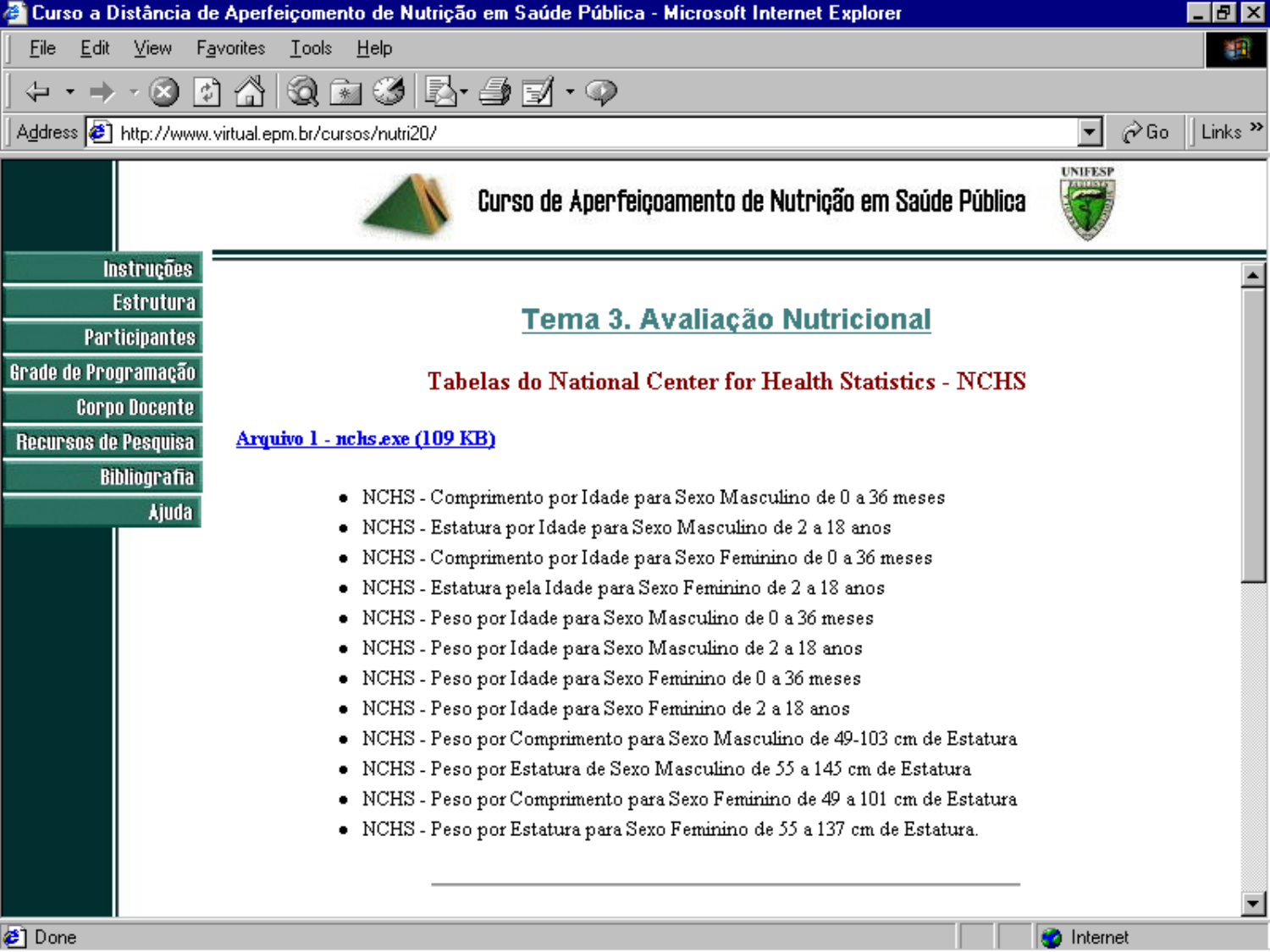

## Slide 17
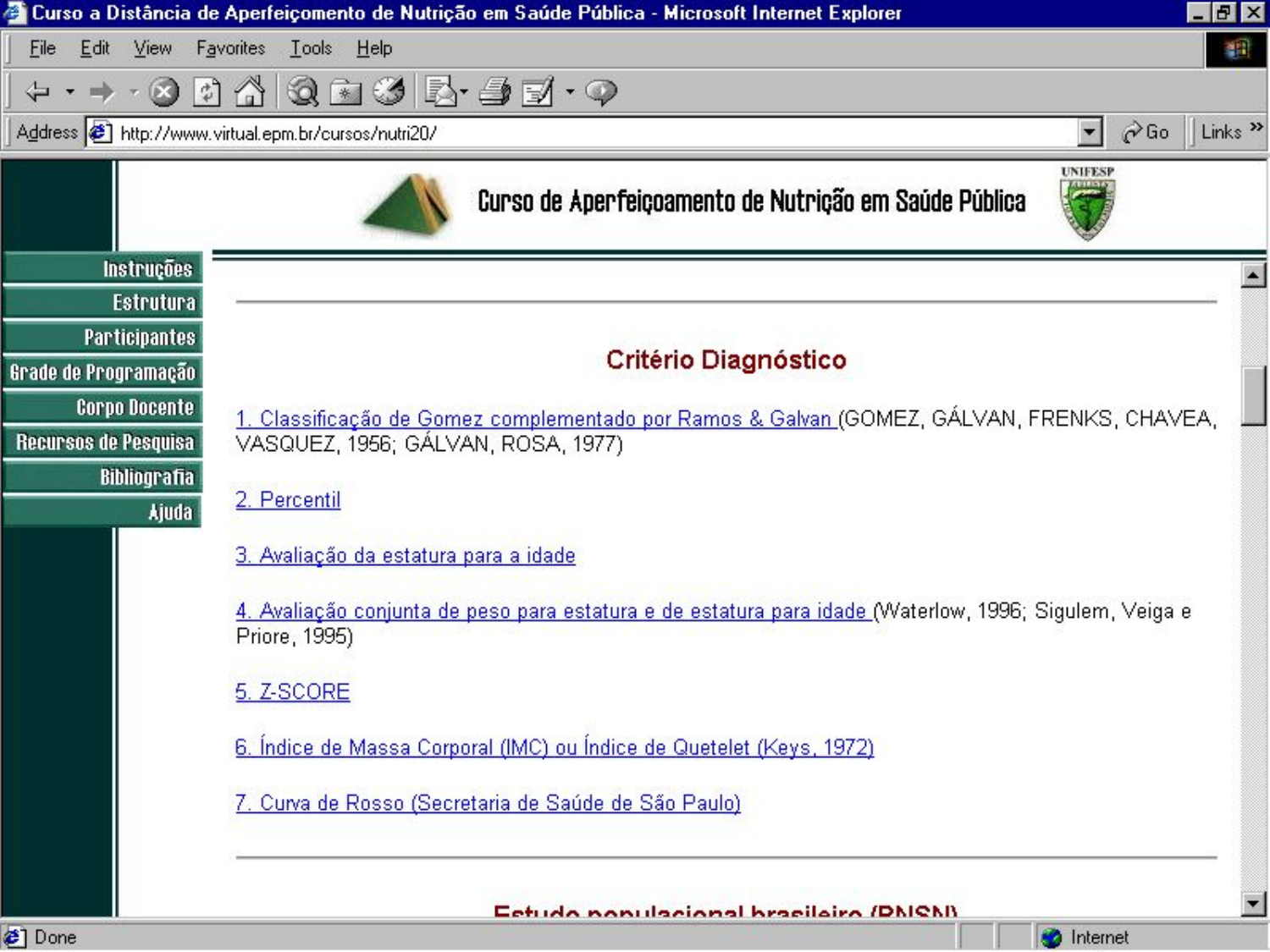

## Slide 18
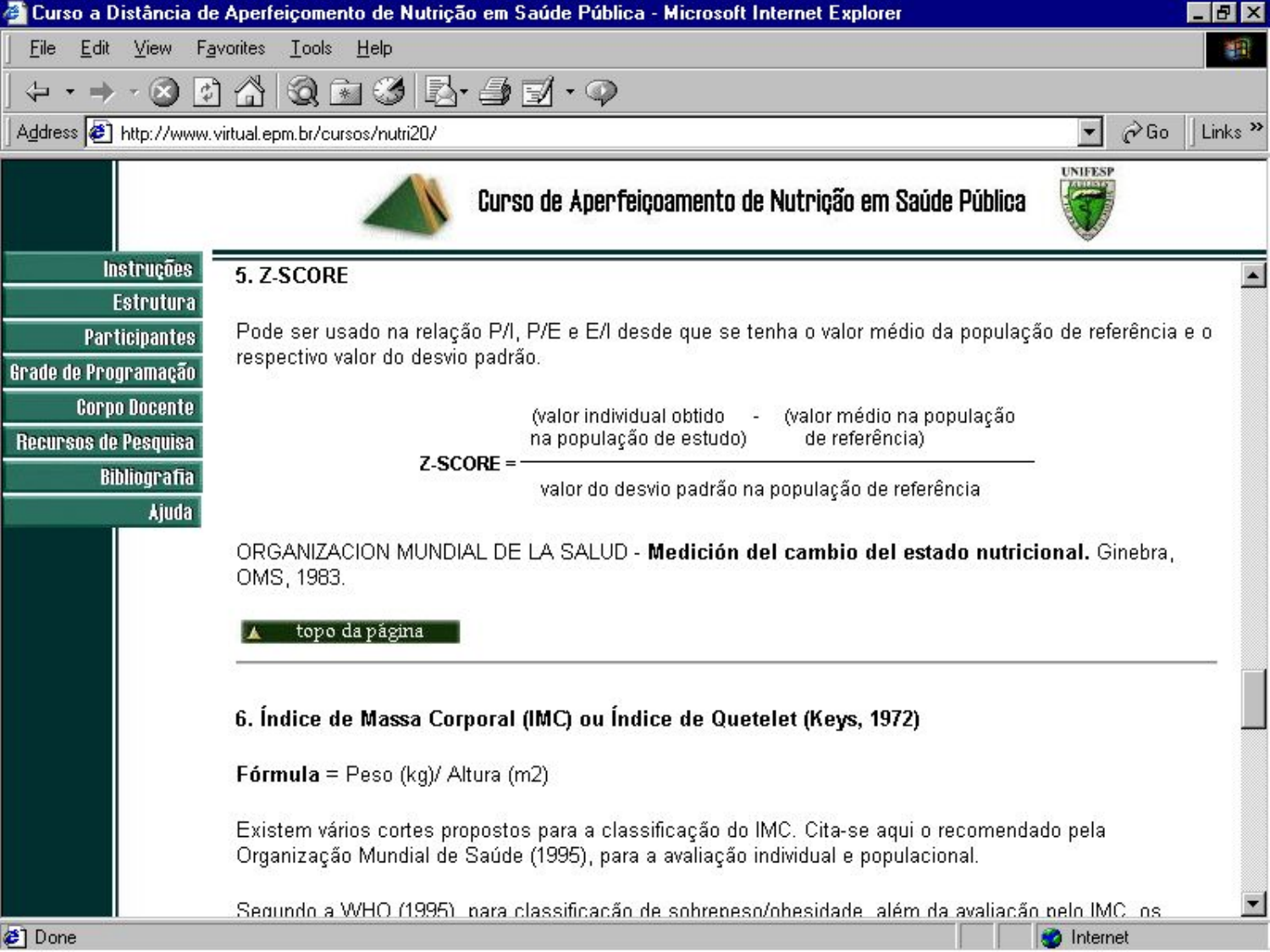

## Slide 19
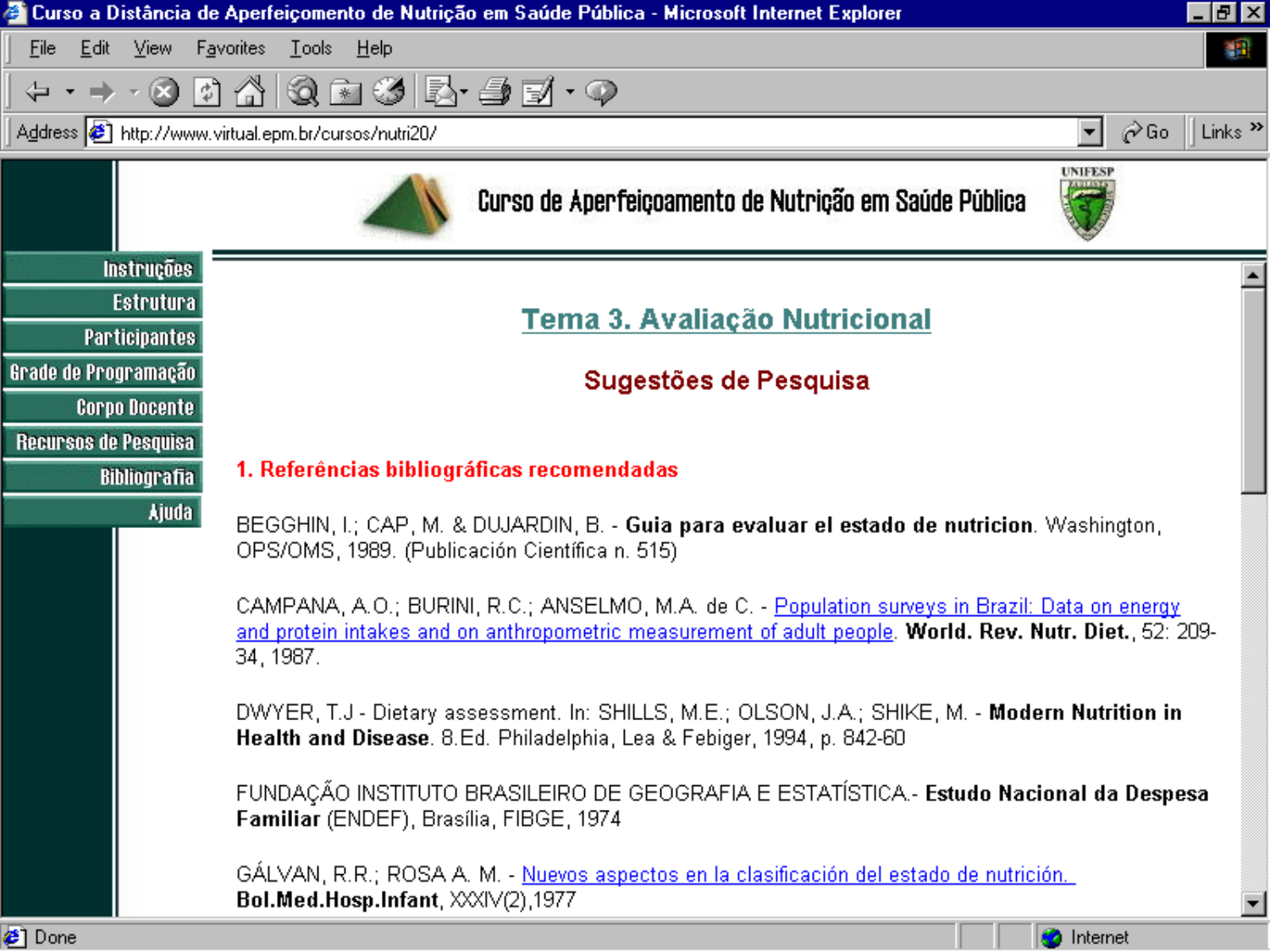

## Slide 20
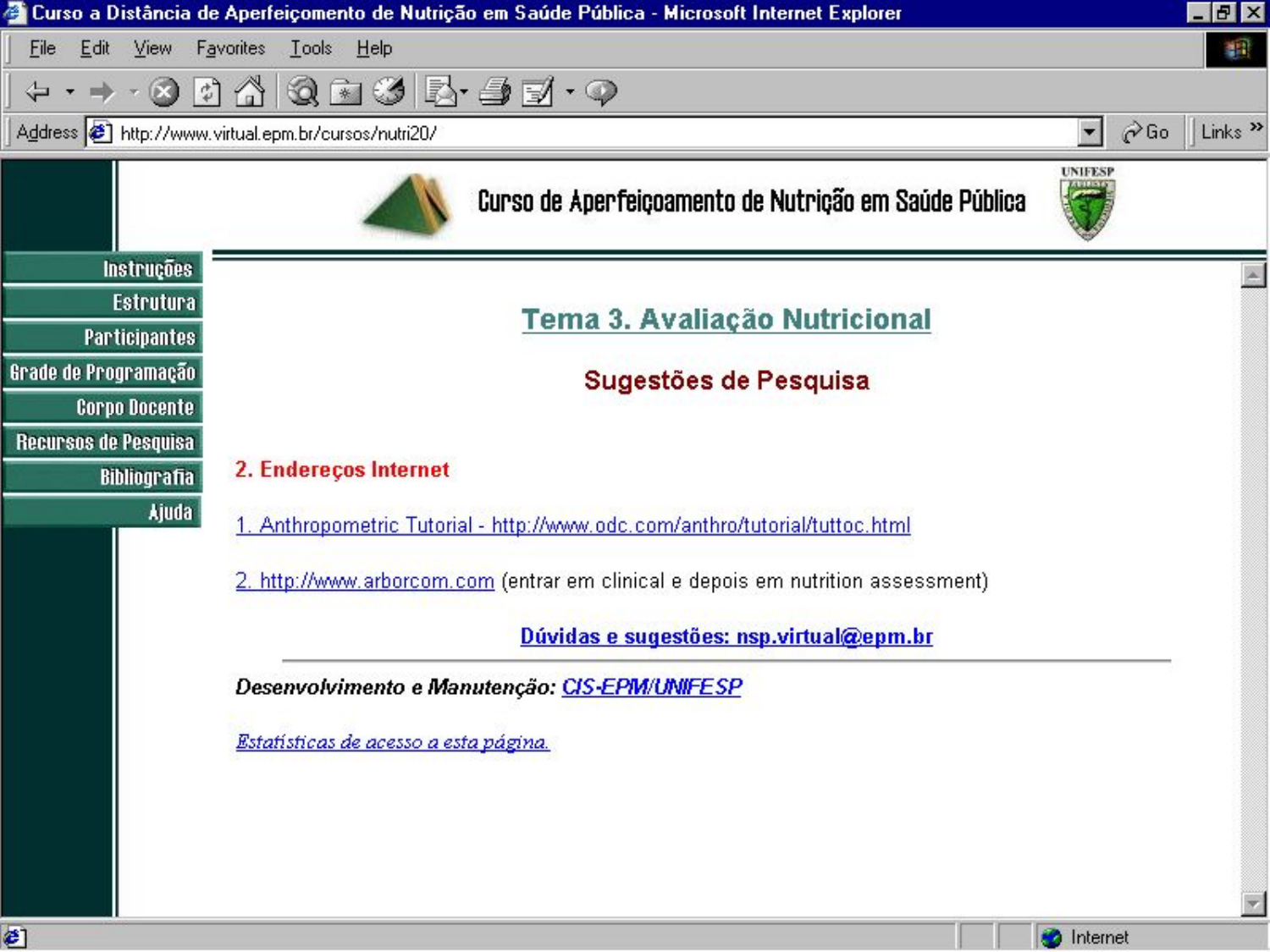

## Slide 21
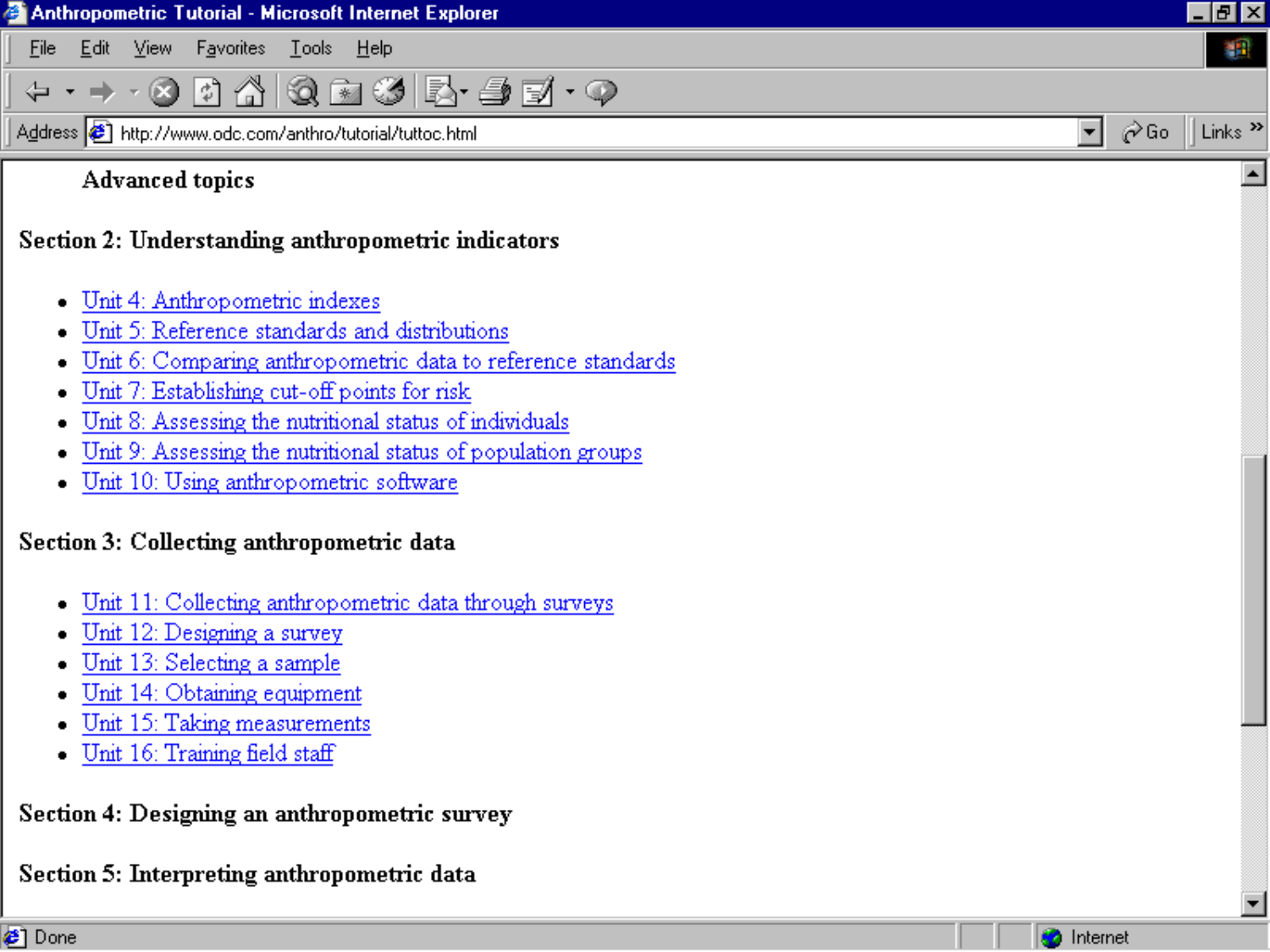

## Slide 22
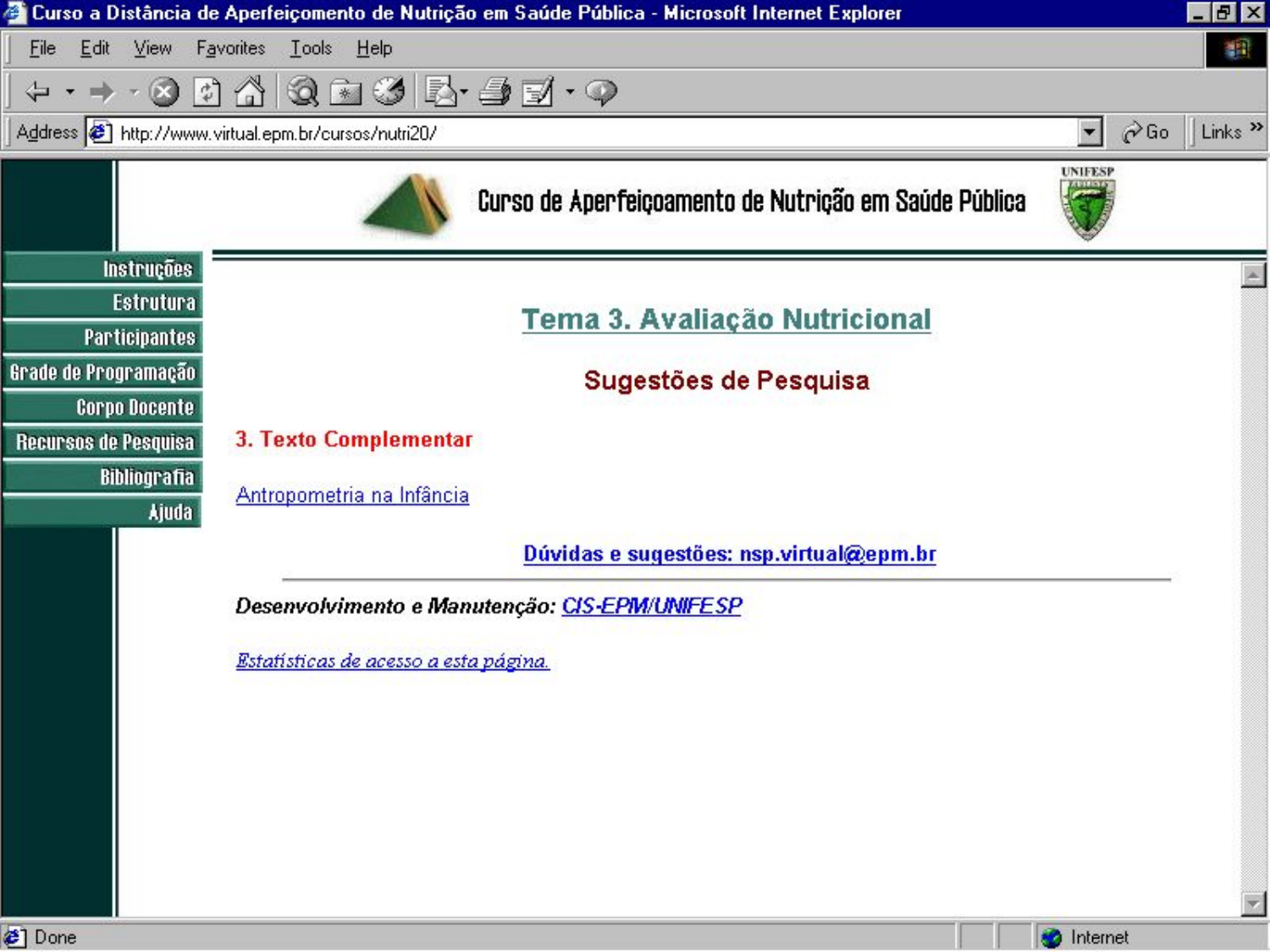

## Slide 23
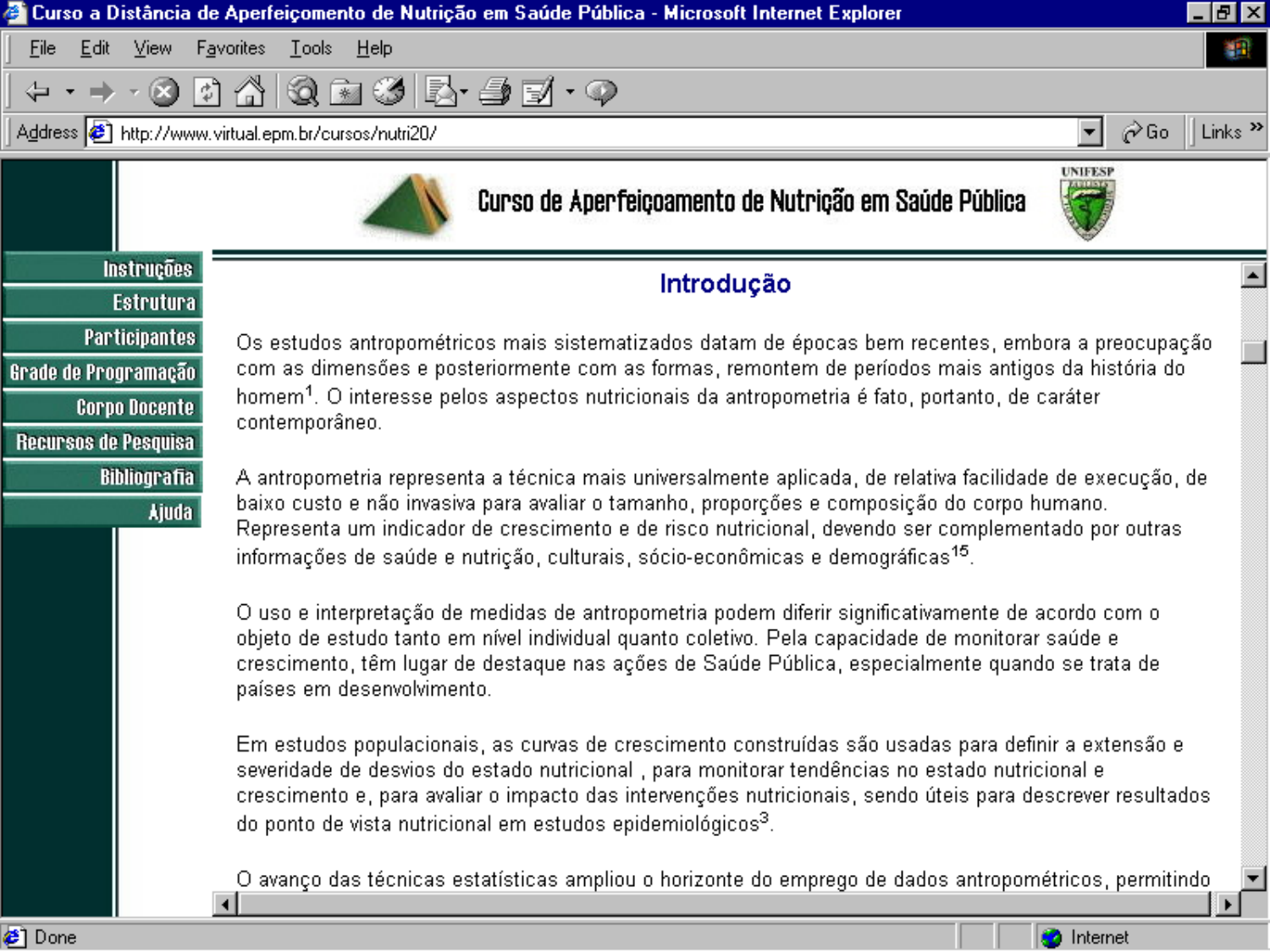

## Slide 24
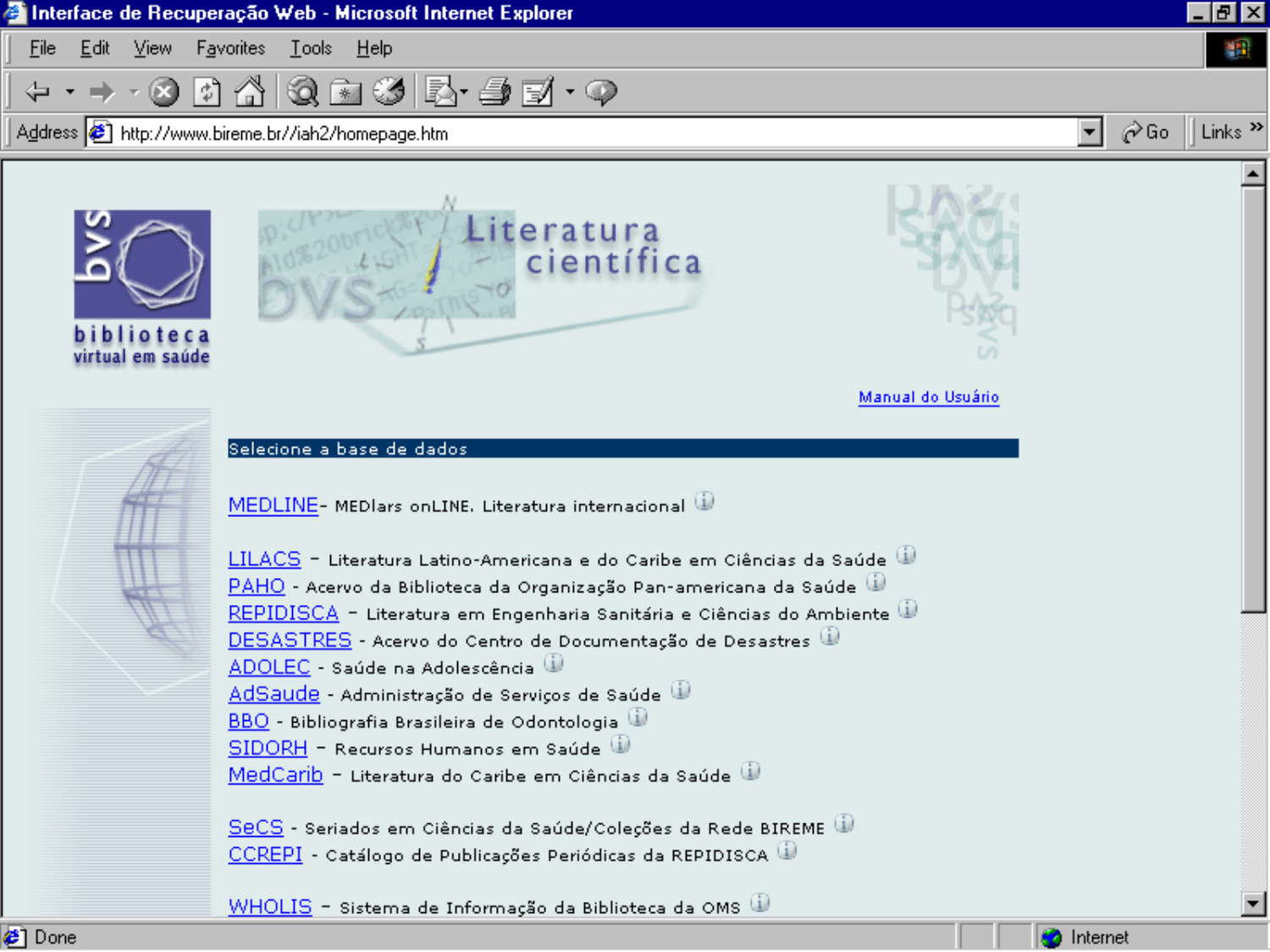

## Slide 25
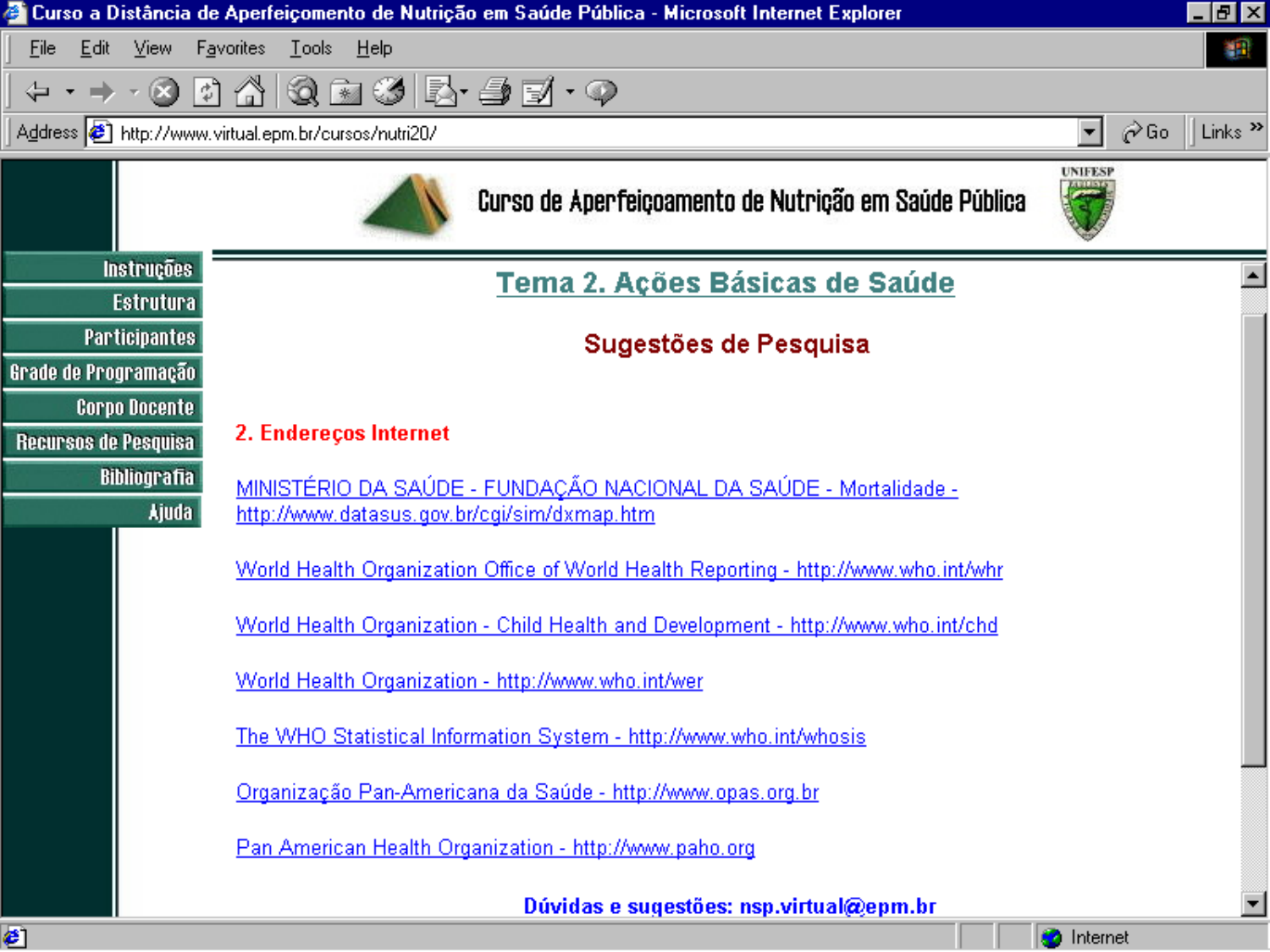

## Slide 26
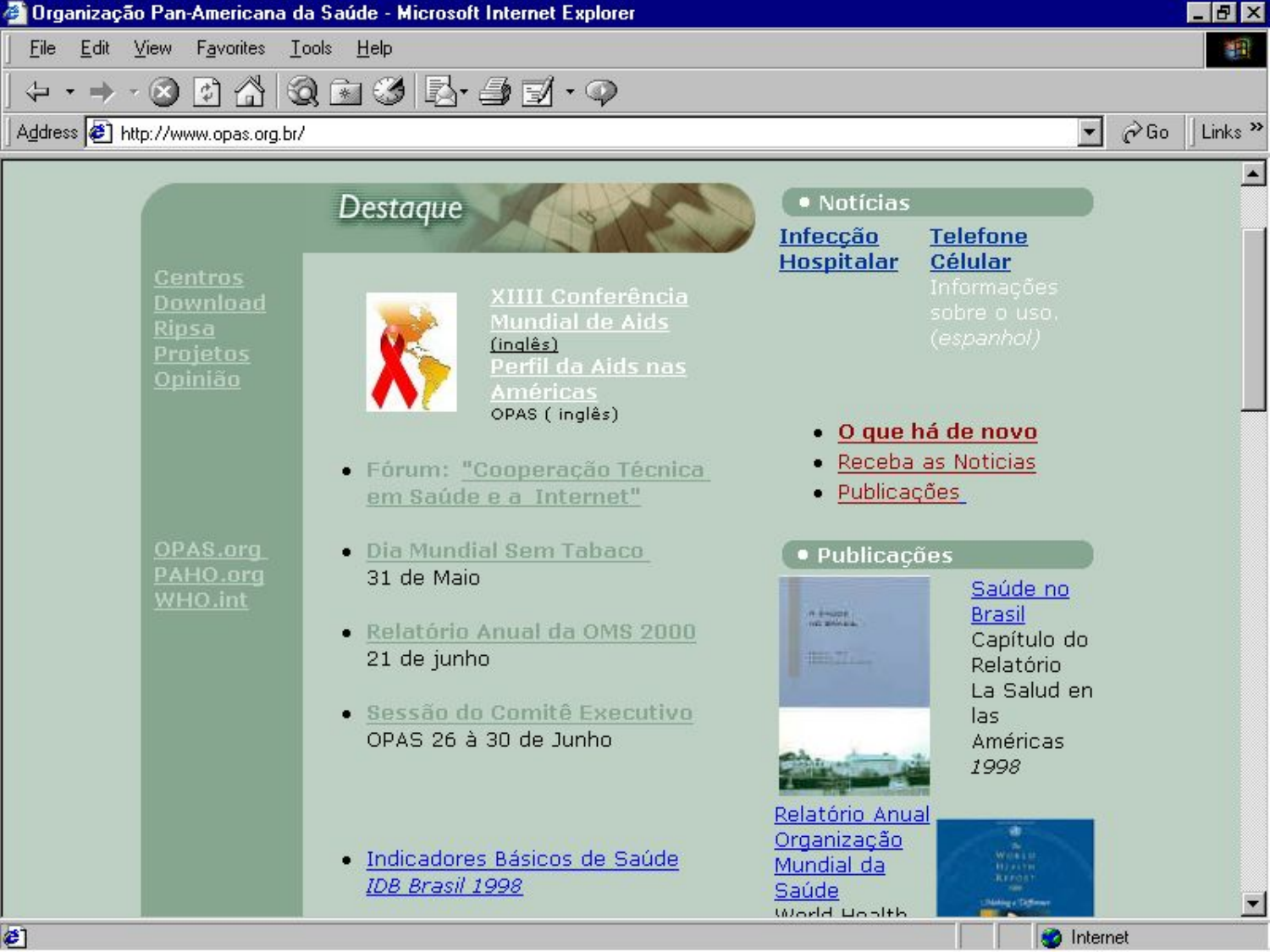

## Slide 27
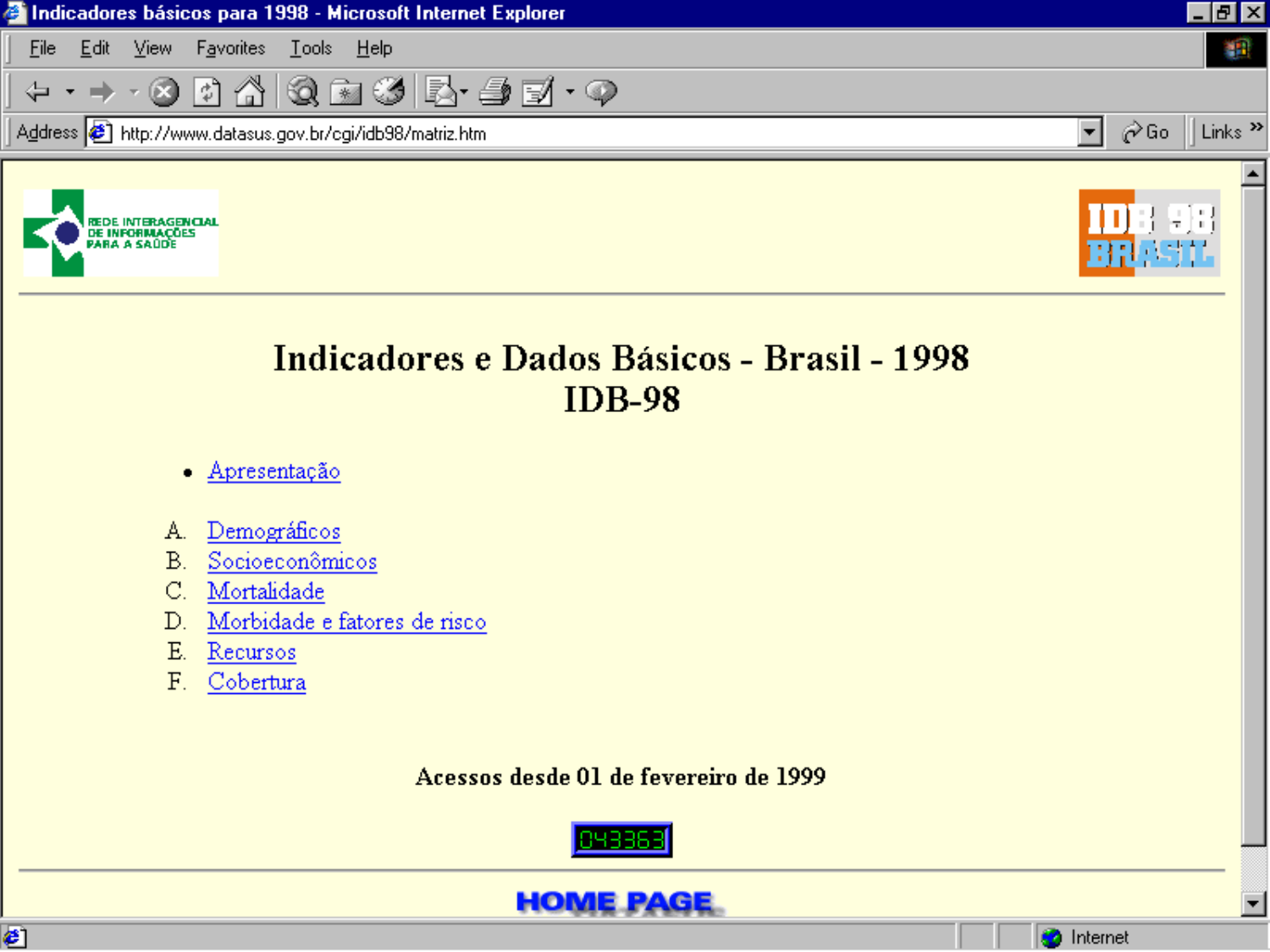

## Slide 28
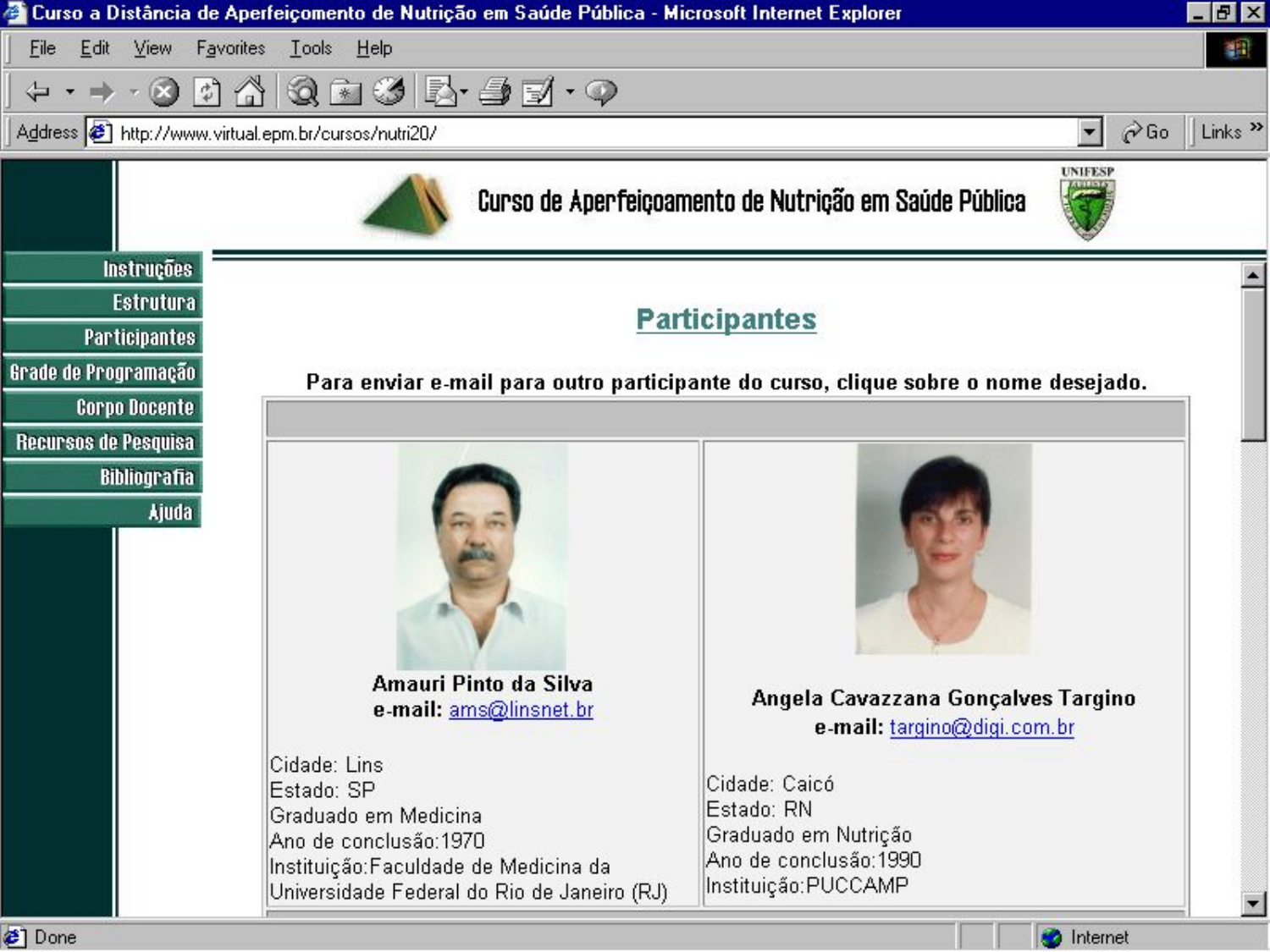

## Slide 29
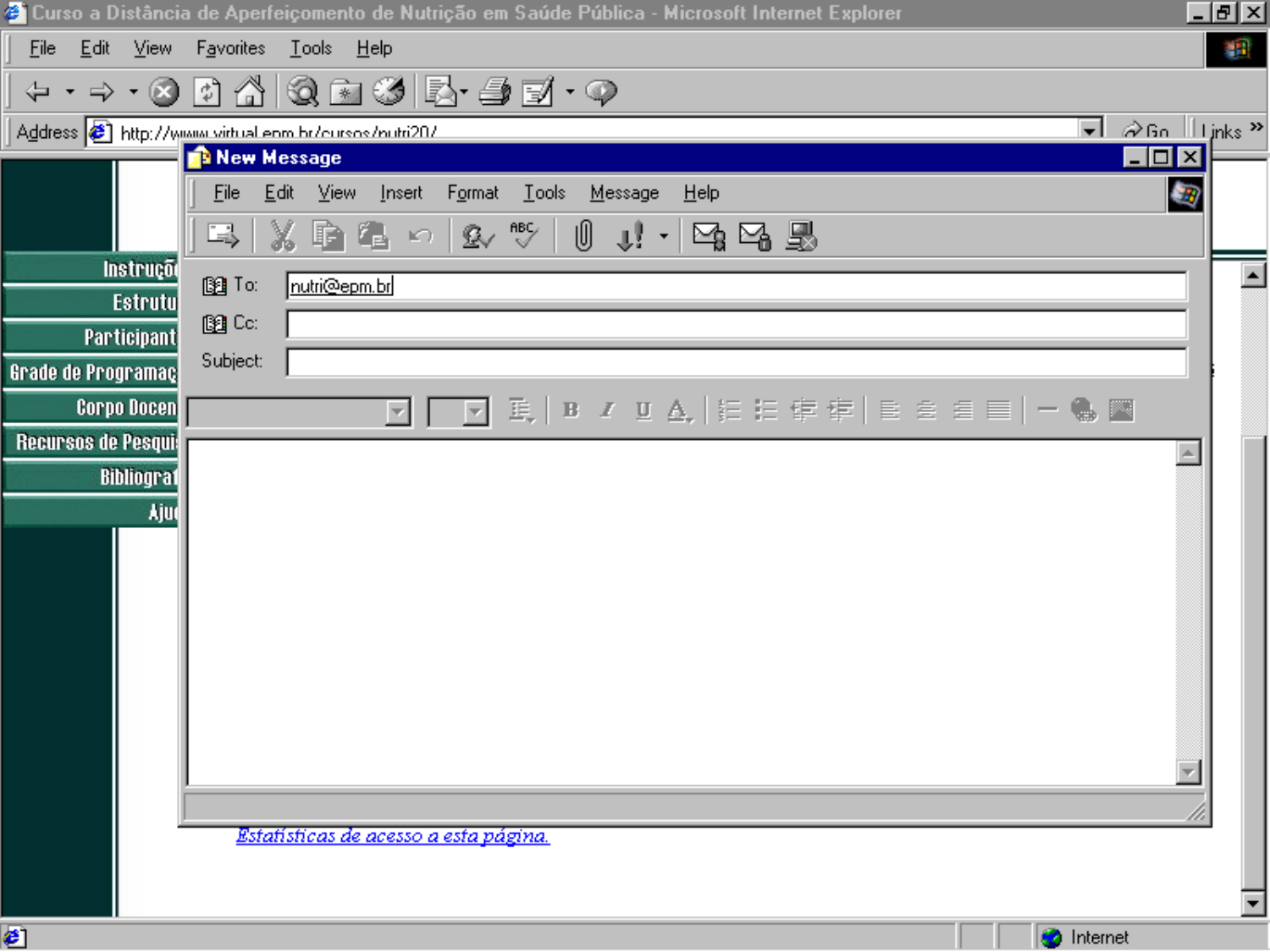

Supplement: Supplementary file 1 [file jmir_v3i2e16_app1.ppt]
